# Single-cell and spatial transcriptomics reveal metastasis mechanism and microenvironment remodeling of lymph node in osteosarcoma

**DOI:** 10.1186/s12916-024-03319-w

**Published:** 2024-05-17

**Authors:** Yun Liu, Mingwei He, Haijun Tang, Tianyu Xie, Yunhua Lin, Shangyu Liu, Jiming Liang, Feicui Li, Kai Luo, Mingxiu Yang, Hongcai Teng, Xiaoting Luo, Juliang He, Shijie Liao, Qian Huang, Wenyu Feng, Xinli Zhan, Qingjun Wei

**Affiliations:** 1 Department of Spine and Osteopathic Surgery, The First Affiliated Hospital of Guangxi Medical University, Nanning, Guangxi 530021 China; 2 Department of Traumatic Orthopedic and Hand Surgery, The First Affiliated Hospital of Guangxi Medical University, Nanning, Guangxi 530021 China; 3 Department of Pharmacy, The First Affiliated Hospital of Guangxi Medical University, Nanning, Guangxi 530021 China; 4 Department of Bone and Soft Tissue Tumor, Guangxi Medical University Cancer Hospital, Nanning, Guangxi 530021 China; 5 Guangxi Key Laboratory of Regenerative Medicine, Orthopedic Department, The First Affiliated Hospital of Guangxi Medical University, Nanning, 530021 China; 6 Department of Bone and Joint Surgery and Sports Medicine, The Second Affiliated Hospital of Guangxi Medical University, Nanning, 530007 China

**Keywords:** Spatial transcriptomics, Single-cell transcriptomics, IBSP, Osteosarcoma

## Abstract

**Background:**

Osteosarcoma (OS) is the most common primary malignant bone tumor and is highly prone to metastasis. OS can metastasize to the lymph node (LN) through the lymphatics, and the metastasis of tumor cells reestablishes the immune landscape of the LN, which is conducive to the growth of tumor cells. However, the mechanism of LN metastasis of osteosarcoma and remodeling of the metastatic lymph node (MLN) microenvironment is not clear.

**Methods:**

Single-cell RNA sequencing of 18 samples from paracancerous, primary tumor, and lymph nodes was performed. Then, new signaling axes closely related to metastasis were identified using bioinformatics, in vitro experiments, and immunohistochemistry. The mechanism of remodeling of the LN microenvironment in tumor cells was investigated by integrating single-cell and spatial transcriptomics.

**Results:**

From 18 single-cell sequencing samples, we obtained 117,964 cells. The pseudotime analysis revealed that osteoblast(OB) cells may follow a differentiation path from paracancerous tissue (PC) → primary tumor (PT) → MLN or from PC → PT, during the process of LN metastasis. Next, in combination of bioinformatics, in vitro and in vivo experiments, and immunohistochemistry, we determined that ETS2/IBSP, a new signal axis, might promote LN metastasis. Finally, single-cell and spatial dissection uncovered that OS cells could reshape the microenvironment of LN by interacting with various cell components, such as myeloid, cancer-associated fibroblasts (CAFs), and NK/T cells.

**Conclusions:**

Collectively, our research revealed a new molecular mechanism of LN metastasis and clarified how OS cells influenced the LN microenvironment, which might provide new insight for blocking LN metastasis.

**Supplementary Information:**

The online version contains supplementary material available at 10.1186/s12916-024-03319-w.

## Background

Osteosarcoma, which is prevalent in children and adolescents, is the most common primary malignant bone tumor [1, 2]. Osteosarcoma is very prone to metastasis, and the prognosis of patients with metastasis is extremely poor [3, 4]. Main metastasis manners include hematogenous metastasis and lymphatic metastasis. Compared with hematogenous metastasis, patients with LN metastasis present a worse prognosis with a survival rate of only 10.9% [5–7]. Thus, an exhaustive analysis of the molecular mechanism of LN metastasis in osteosarcoma is of great significance to improve the prognosis of patients.

LN metastasis is a continuous pathological process. On the one hand, the prerequisite of metastasis is the invasion of tumor cells into LN. Research suggests that cancer cells can actively migrate towards and invade lymphatic vessels by sensing chemokine gradients produced by lymphatic endothelial cells (LECs) [8]. Chemokines like CXCL10, CXCL12, CCL1, CCL19, and CCL21, produced by LECs, can be detected by tumor cells with matching receptors, prompting their attraction towards lymphatic vessels. Additionally, tumor cells and associated stromal cells can secrete factors such as VEGF-C and VEGF-D, which induce lymphangiogenesis by activating VEGFR-3 in LECs, thereby creating more pathways to lymphatic vessels. This phenomenon allows cancer cells to actively access lymphatic vessels for metastasis [9]. However, most of the studies on LN metastasis in osteosarcoma are clinical case studies [10]. They have confirmed the hazard of LN metastasis, but few reports have focused on the metastasis mechanism. On the other hand, the remodeling of the LN microenvironment by tumor cells is another important content. Studies have shown that tumor cells will reconstruct the immune landscape of LN after planting, which was beneficial for the growth of tumor cells. In MLN of breast cancer, for example, tumor cells can inhibit the activity of NK cells to reduce cytotoxicity, thus promoting their own survival and growth [11]. However, the mechanism by which tumor cells remodel the microenvironment of LN has not been reported in osteosarcoma.

Here, for the first time, we profile paracancerous, primary tumor, and LN via single-cell RNA (scRNA) sequencing, identify a subpopulation of malignant osteoblastic cells, that is OS cells, which exert metastasis function, and determine a new signal axis closely related to metastasis using bioinformatics, in vitro experiments and immunohistochemistry. Besides, the issue of how tumor cells remodel the microenvironment of LN is also investigated via integrating single-cell and spatial transcriptomics. This study would provide new and deeper insights into the LN metastasis of OS cells and explore the remodeling process of the LN microenvironment, which will provide a new theoretical basis for preventing lymph node metastasis in osteosarcoma patients.

## Methods

### Clinical sample collection

Eleven patients treated at the First Affiliated Hospital of Guangxi Medical University between November 2019 and December 2023 were included in this study. Inclusion and exclusion criteria were developed as follows: inclusion criteria: (1) confirmed diagnosis of osteosarcoma by postoperative histopathology; (2) lesions located in the extremities; exclusion criteria: (1) comorbid with other diseases such as fever, infection, and lymph node disease; (2) recurrent patients. A total of 14 tissue samples were finally collected from the eight patients, including eight PT, four PC, and two LN. Among the 8 primary cancer tissues, 6 cases have been reported in our previous study (accession number GEO: GSE162454) [12]. Detailed clinical information is provided in Table S1 (Additional file 1). All patients were informed and signed informed consent for this study, which was approved by the Ethics Committee of the First Affiliated Hospital of Guangxi Medical University (ethics number: 2019KY-E-097).

### Preparation of single-cell suspensions

Immediately after surgical excision to remove the tumor, we collected fresh samples and placed them in an ice solution containing Hank’s balanced salt solution and 1% antifungal antibody and transferred the tissue into a 4 ℃ refrigerator for storage within 20 min. Subsequently, a small soft tissue sample was taken and cut into 1 mm^3^ pieces, washed well using Dulbecco’s phosphate-buffered saline (DPBS) (cat. no. C14190500BT; Thermo Fisher Scientific, Inc.), and digested by adding collagenase 2 (1 mg/mL) for 45 min at 37 ℃. The cells were filtered using a 100-μM cell filter, and the obtained cell suspension was centrifuged for 5 min at 300 g. The supernatant was removed, and red blood cells were removed using 1× red blood cell lysis buffer (10× diluted to 1×; cat. no. B250015; BioLegend, Inc.). Finally, after washing the cells with Phosphate-Buffered Saline (PBS), the cells were resuspended using DPBS. Meanwhile, cell viability will be assessed using 0.4% Trypan blue staining.

Paracancerous tissues contain more hard bone composition, which presents great challenges in single-cell sequencing. To overcome this limitation, in this study, we first used αMEM (SH30265.01, HyClone) to clean the hard bone three times and bite them into small pieces of about 1–2 mm in diameter. Twelve milliliters of collagenase type II at a concentration of 2 mg/mL (Cat: A004174-0001, Sangon Biotech) was mixed with αMEM containing 100 U/ mL penicillin and 100 μg/mL streptomycin (Cat:15140-122, Gibco) in a 50-mL conical tube. The bone snap was then placed in it and digested at 37 °C for 25 min with gentle agitation. Afterwards, the collagenase solution was removed aseptically and the bone pieces were rinsed three times in 10 mL of PBS. After five rounds of digestion, the collagenase solutions from the last two rounds of digestion were combined and the solutions were filtered through a 40-μm filter. Finally, red blood cell lysis solution (10×) was used to remove red blood cells according to the manufacturer’s instructions.

### Single-cell library creation and sequencing

The individual cells obtained from each sample were loaded onto a 10× genomic single-cell chip. Samples were processed using the 10× Genomics V3 Barcode Chemistry Kit according to the manufacturer’s instructions. The mRNA from the droplets was subjected to a reverse transcription reaction, followed by cDNA amplification. The single-cell libraries were then sequenced on an Illumina HiSeq X Ten instrument (Illuminia, Inc.).

### Quantification of single-cell gene expression

Sample demultiplexing, barcode processing, and single-cell 3′ counting were performed using Cell Ranger software (version 2.2.0; 10× Genomics, USA). “mkfastq” function in Cell Ranger was used to generate the raw files into sample-specific fastq files. Subsequently, the fastq files of each sample were subjected to alignment against the human reference genome (GRCh38) using the “count” function within Cell Ranger, enabling the quantification of gene expression levels at the single-cell level.

### Quality control and batch correction

Quality control of cells was performed using the Seurat package (version 4.3.0; https://satijalab.org/seurat/install.html), and the cells with nFeature_RNA < 500, nFeature_RNA > 6500, mitochondrial genes > 10%, and erythrocyte genes > 1% were defined as low-quality cells. After quality control, a total of 78,609 high-quality cells were obtained. Additionally, we employed the Harmony package (version 0.1.1; https://github.com/immunogenomics/Harmony) to integrate and remove batch effects from sequencing data across different tissues and patients.

### Cell clustering

The clustering of the overall cells was primarily achieved through the implementation of the “RunUMAP” function (with parameter selection reduction = “harmony”, dims = 1:30), “FindNeighbors” function (with parameter selection reduction = “harmony”, dims = 1:30), and “FindClusters” function (with resolution = 0.3) in the Seurat package. The “FindAllMarkers” function was employed to identify genes exhibiting specific expression patterns in each cell cluster.

### CNV analysis

To determine the variation in copy numbers of OB cells, we employed copy number variation (CNV) analysis via the inferCNV package (version 1.2.1). OB cells from PC were regarded as the reference for alignment, and the variation of gene expression intensity on each chromosome of OB cell was calculated. For the selection of parameters, denoise = TRUE, analysis_mode = “subcluster”, and cutoff = 0.1 were used.

### Search for differential genes and GSVA analysis

We extracted OB cells from all the cells using the subset function and obtained 7 subgroups after dimensional reduction and clustering. GSVA analysis was used to perform pathway enrichment analysis based on all available genes. GSVA package (version 1.46.0) with the “gsva” function was used to perform GSVA and C5 gene sets and hallmark gene sets from the MSigDB database (http://www.gsea-msigdb.org/gsea/index.jsp) were selected as reference geneset.

### Survival analysis of differential genes based on bulk RNA sequencing

First, we utilized the “FindAllMarkers” function to identify genes highly expressed in C5 compared to other clusters in LN, as well as genes highly expressed in the LN compared to PT. The intersection of these two sets was regarded as genes that may contribute to the metastasis of C5 cells. Subsequently, we obtained the expression matrix of osteosarcoma bulk RNA sequencing data from the UCSC database (http://xena.ucsc.edu/). Clinical and prognostic information of patients was downloaded from the TARGET database (https://ocg.cancer.gov/programs/target). After integrating the information, we excluded data from 4 cases with incomplete clinical information, resulting in the inclusion of 84 patients’ bulk RNA sequencing data for further analysis. Lastly, the differential genes were subjected to univariate Cox regression using the survival package, with *p* < 0.05 indicating genes with survival significance.

### Cell-cell communication analysis

CellPhoneDB is a publicly available database that provides curated annotations of receptors, ligands, and their interactions. In our study, we employed CellphoneDB.py (version 0.22) to compute the strength and likelihood of receptor-ligand interactions between the C5 group and NKT cells, CAFs, B cells, and myeloid cells. Receptor-ligand pairs with interaction strength values greater than 1 and *p* < 0.05 were considered potential signaling axes between the two cell populations.

### Pseudotime analysis

To investigate the evolutionary process of OB cells during tumor metastasis, we conducted pseudotime analysis using Monocle 2. First, we normalized the expression of cell mRNA using the “estimateSizeFactors” function. Then, we evaluated the data dispersion using the “estimateDispersions” function. Subsequently, we performed PCA analysis and clustering on the data using the default settings of the Monocle2 package. We identified differential genes using the “differentialGeneTest” function and selected the top 1000 differentially expressed genes for developmental trajectory ordering. Finally, we employed DDRTree for dimensionality reduction and visualized the results using the “plot_cell_trajectory” function.

### Subgroup metastasis scoring

Genes potentially mediating osteosarcoma metastasis were compiled based on previous literature reports (Additional file 2: Table S2). The “AddModuleScore” function from the Seurat package and the calcAUC function of the AUCell package were then employed. Corresponding scores and AUC values for different subgroups were generated based on genes related to metastasis, allowing the observation of the correlation of each subgroup with metastasis.

### Prediction of transcription factors

The gene sequence of integrin binding sialoprotein (IBSP) was obtained from the UCSC database (https://genome.ucsc.edu/). We designated the transcription factor binding region of the gene as +2000 ~ −100. The PROMO database (https://alggen.lsi.upc.es/cgi-bin/promo_v3/promo/promoinit.cgi?dirDB=TF_8.3) was then used for predicting transcription factors.

### Sample preparation for lymph node spatial transcriptomics

After tissue dissociation, residual blood and free tissue on the tissue surface were thoroughly washed with PBS solution and placed into a 15-mL centrifuge tube containing MACS Tissue Storage Solution (Miltenyi, catalog no. 130-100-008). The aforementioned steps were performed under aseptic conditions and completed within 30 min to prevent RNA degradation. The samples were then snap-frozen in optimum cutting temperature compound (Sakura, catalog no. 4583) and stored at – 80 °C prior to cryosectioning. To obtain the tumor regions within the lymph nodes, a series of sectioning and hematoxylin and eosin (HE) staining were performed with the assistance of a pathology expert. To include a broader range of tissue components, the peripheral regions of the lymph nodes were ultimately selected. The sections were cut at a size of 6.5 mm × 6.5 mm with a thickness of 10 μm.

### Tissue permeabilization, library construction, and sequencing

For tissue permeabilization, the slides were first placed in the Slide Cassette (from the Visium Slide kit) for the optimal permeabilization time. In this study, all the tissue sections were permeabilized for 12 min. A permeabilization enzyme (from the Visium Reagent kit) was used for permeabilizing the tissue sections on the slide for incubating for the predetermined permeabilization time. The polyadenylated mRNA released from the overlying cells was captured by the primers on the spots. Library construction was performed with a Library Construction Kit (10 × Genomics, catalog no. PN-1000190). The barcoded libraries were sequenced using the Illumina Nova Seq 6000 platform with the PE150 sequencing mode.

### Spatial transcriptome data analysis

The expression data processing was performed using Space Ranger, which involved demultiplexing, alignment to the hg38 human reference genomes, tissue detection, landmark detection, and barcode/UMI counting. Initially, cells of low quality were filtered out based on quality control conditions: minCount = 1500, minFeature = 500, and maxMT = 25. To correct the sequencing depth of high-quality spots, SCTransform from Seurat was used for standardization and identification of highly differentially expressed genes. PCA was employed for dimensionality reduction and comparison of similarities between spots. The first 30 principal components of each spot were used for graph-based clustering analysis, and the identified clusters were visualized using UMAP. Based on the spot clustering results from Seurat, the FindAllMarkers function was used to identify differentially expressed genes between distinct spot clusters. Genes meeting the criteria of *p* value ≤ 0.05 and log2FC ≥ 1.5 were considered as marker genes for a cluster compared to all remaining clusters. In this study, we employed a mapping method combined with the AddModuleScore approach to annotate cell types. Firstly, FindTransferAnchors was used to identify corresponding “anchors” in the single-cell Seurat object. Then, the TransferData command was applied to calculate which groups in the scRNA might correspond to the subgroups in the spatial transcriptomics data. Additionally, using the AddModuleScore method, we scored each spatial subgroup based on the top 50 feature genes from scRNA data. By integrating the results of these two methods, we finally determined the cell types of each spatial subgroup.

### Cell culture

Human osteosarcoma cells (Saos2, 143B) and normal osteoblast cell line (hFOB 1.19) were provided by the Procell Company (Wuhan, China). Saos2 cells were cultured in McCoy’s medium supplemented with 15% FBS, while 143B cells were in RPMI 1640 medium supplemented with 10% FBS. hFOB cells were cultured in DMEM/F-12 medium. All osteosarcoma cell lines were maintained in a humidified incubator at 37 °C with 5% CO_2_, while hFOB 1.19 cells were cultured at 35 °C with 5% CO_2_.

### Cell transfection

143B and Saos2 cell lines were transfected according to the manufacturer’s protocol, and stably transfected cell lines were generated through puromycin selection after passaging. Transfection efficiency was assessed by detecting the fluorescent density provided by the virus, as well as the relative expression levels of IBSP and ETS Proto-Oncogene 2 (ETS2). The si-IBSP, si-ETS2 vectors, and empty vector (Vector) lentiviral plasmids were constructed by Sangon Biotech (Shanghai) Company. The siRNA sequences are provided in Table S3 (Additional file 3).

Saos2 and 143B cell lines were transfected with lentiviral overexpression vectors to establish stable cell lines overexpressing IBSP. Lentivirus was purchased from Sangon Biotech, Shanghai, China. Saos2 and 143B cells were inoculated in 6-well plates with 200,000 cells per well, respectively, and set up with oe-N.C. (overexpression negative control group) and oe-IBSP group (overexpression IBSP group). When SAOS and 143B cells were adhered to the wall and achieved a confluency of over 50%, they were transfected using lentivirus. The multiplicity of infection (MOI) of the cells was set at 60. After 24 h post-transfection, complete medium containing lentivirus was removed and replaced by new complete medium. The cells were then incubated for a total of 72 h and subsequently will be observed under an inverted fluorescence microscope. Upon reaching 80% transfection efficiency in Saos2 and 143B cells, puromycin (2 μM) was used to eliminate cells that were not successfully transfected. Subsequently, the cells were transferred to T25 culture flasks for expansion. Finally, qRT-PCR was employed to confirm whether the stably transfected cell lines overexpressed IBSP.

### QRT-PCR experiment

RNA extraction was performed using the Hipure Total RNA Mini Kit (Magen, China), followed by reverse transcription using the cDNA synthesis kit (Takara, Japan). Subsequently, RT-qPCR was carried out using SYBR Green (FastStart Universal SYBR Green Master ROX, Germany). GAPDH was used as a reference gene for normalization, and the 2^–ΔΔCt^ method was employed to calculate the RNA expression levels. The primer sequences are provided in Table S4 (Additional file 4).

### EDU cell proliferation assay

The transfected Saos2 and 143B cells were subjected to a proliferation assay using the BeyoClick™ EdU-488 cell proliferation assay kit (C0071S, Beyotime, Shanghai). Briefly, the cells were seeded in a 96-well plate. They were then incubated with EDU solution (50 μl per well) for 2 h, followed by fixation with 4% paraformaldehyde for 15 min. The cells were subsequently washed twice with alternating blocking and permeabilization solutions for 5 min each and then incubated with the click staining solution for 30 min in the dark. Finally, the click staining solution was removed, and the cells were washed and incubated with Hoechst solution for 10 min, also in the dark. After removing the Hoechst solution, the cells were washed three times. Images were immediately captured using an inverted fluorescence differential interference contrast microscopy system (cellSens Dimension, OLYMPUS).

### Transwell invasion assay

Matrigel matrix gel (356234, Corning, USA) was dissolved overnight at 4 °C and diluted in serum-free medium at a ratio of 1:8. Subsequently, 50 μL of the diluent was added to the middle of the membrane at the bottom of the upper chamber base. Saos2 and 143B cells (5 × 104/well) transfected with si-NC, si-IBSP, and si-ETS2 were inoculated into the upper chamber, and 600 µl of culture medium containing 10% FBS was added to the lower chamber. After 48 h of incubation at 37 °C, the cells were fixed with 4% paraformaldehyde for 15 min. The cells on the upper side of the membrane were wiped off with a cotton swab, followed by staining with 0.1% crystal violet for 10 min at room temperature. Five randomly selected microscopic fields were counted using an upright fluorescence differential interference contrast microscopy system (cellSens Dimension, OLYMPUS). The number of cells that invaded the lower chamber was evaluated to assess the invasive ability of tumor cells.

### Immunohistochemical staining

Postoperative pathological slides were collected from 46 osteosarcoma patients treated and pathologically diagnosed in our institute from January 2016 to June 2020. The slides were placed in a 65 °C oven overnight for baking, followed by immersion in a dewaxing solution to remove the paraffin embedded in the tissue, and then subjected to hydration using a gradient of ethanol. Subsequently, 3% hydrogen peroxide was used at room temperature for 15 min to destroy endogenous peroxidase enzymes on the tissue. The slides were placed in a repair box containing sodium citrate repair solution and then subjected to antigen retrieval in a 100 °C water bath for 20 min. Rabbit-derived anti-IBSP antibody (1:400, Proteintech, China) and anti-ETS2 antibody (1:500, Proteintech, China) were incubated overnight at 4 °C. After washing three times with PBS, the slides were incubated with goat anti-rabbit secondary antibody labeled with horseradish peroxidase (Zhongshan Golden Bridge, China). Subsequently, the DAB Substrate Kit was used for incubation, resulting in visible staining. Finally, the relative expression levels were assessed by calculating the percentage of positive area using ImageJ software. The patients were grouped using the median value, and Kaplan-Meier curves were plotted to evaluate the predictive value of IBSP and ETS2 on prognosis.

### Establishment of osteosarcoma paw pad lymph node metastasis model

Ten female BALB/c nude mice, aged 4 weeks, were purchased from Beijing Spefo Bio-technology Co. Ltd, China. These mice were housed under specific pathogen-free conditions in the Animal Center of Guangxi Medical University. All animals were housed in a well-ventilated room with a 12-h light/dark cycle (room temperature: 25 ± 2 °C, humidity: 50% ± 10%) and given standardized food and water. After a 7-day acclimatization period, the mice were randomly divided into two groups: oe-N.C. and oe-IBSP. The right paw pads of the mice were subcutaneously injected with either stably transfected oe-N.C. or oe-IBSP cells, with each mouse receiving 2 × 10^6^ cells in a total volume of 50 μL. The negative control group was injected with an equivalent volume of culture medium. Mice were executed via injection of excess pentobarbital sodium after 4 weeks. The primary tumors in the paw and inguinal lymph nodes were collected and the volumes and weights were measured separately. The formula for calculating the volume of tumors and lymph nodes is as follows: tumor volume = [length * (width)^2^]/2. Subsequently, the lymph nodes were fixed using 4% paraformaldehyde, and paraffin sections were prepared for HE staining to confirm the presence of metastasis. Finally, the lymph node metastasis positivity rate was calculated. This experiment received approval from the Animal Ethics Committee of Guangxi Medical University, and the establishment of the nude mouse model was conducted in accordance with the ethical guidelines set by the same committee.

## Results

### Single-cell atlas of osteosarcoma

**Fig. 1:**
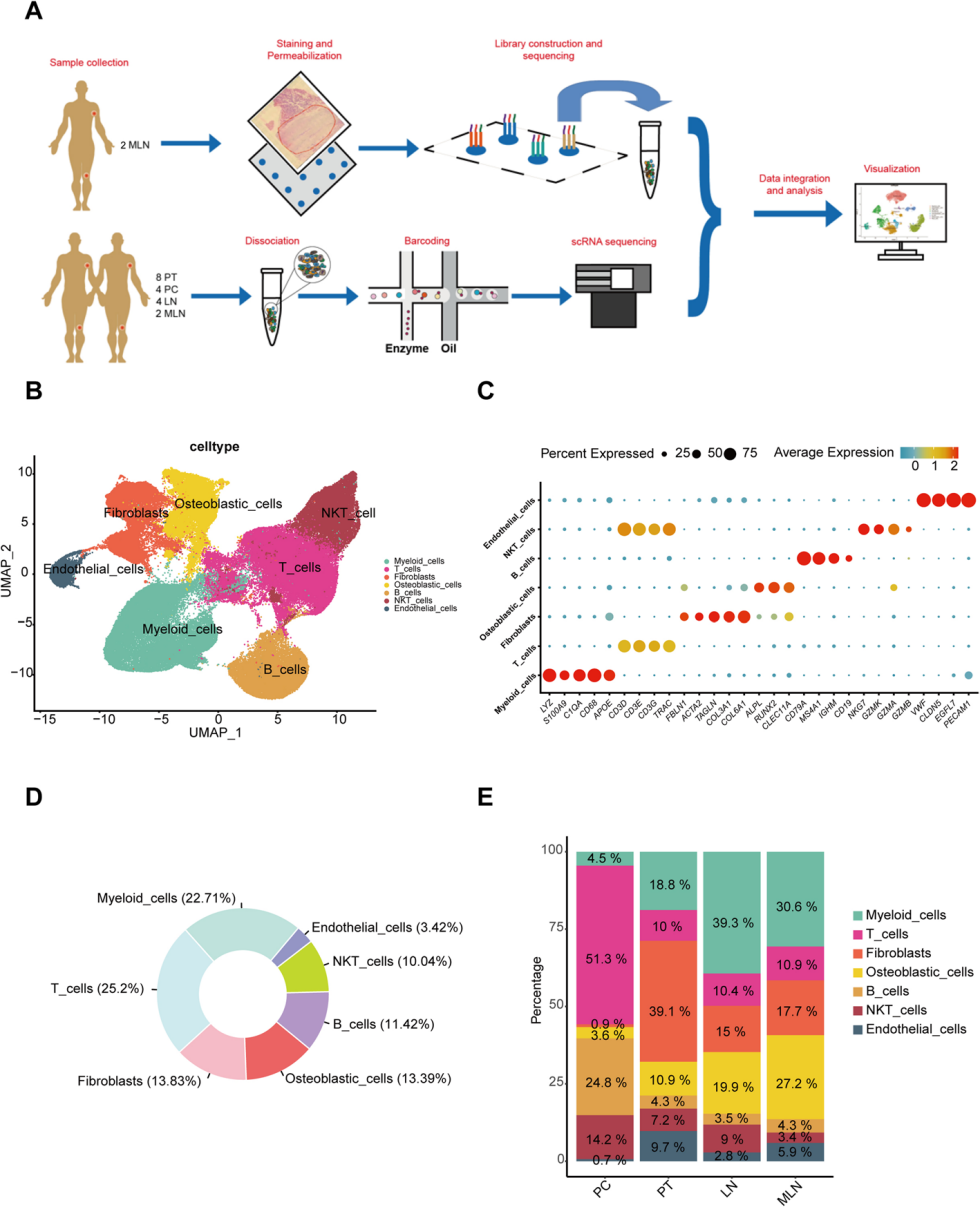
Single-cell atlas of osteosarcoma. **A** The overall flowchart of the research. **B** The UMAP plot of 7 cell clusters from the multicellular ecosystem of 16 tissue samples. **C** The marker gene of each cell cluster. **D** Circular plot of cell proportions for all cell clusters. **E** The composition of cell subpopulations between the different groups

To investigate the process of development and metastasis, 18 samples were collected in this study, including 8 primary tumors (PT), 4 paracancerous (PC), and 6 lymph nodes (LN). In addition, to verify the changes in the microenvironment in the LN, we also performed spatial transcriptome sequencing of two MLN (Fig. 1A). After data quality control, a total of 117,964 cells were finally obtained and classified into 7 cell types (Fig. 1B). The cell populations were annotated according to marker gene expression as follows: myeloid cells (*LYZ*, *S100A9*, *C1QA*, *CD68*, *APOE*); T cells (*CD3D*, *CD3E*, *CD3G*, *TRAC*); fibroblasts (CAFs) (*FBLN1*, *ACTA2*, *TAGLN*, *COL3A1*, *COL6A1)*; osteoblastic cells (OB cells) (*ALPL*, *RUNX2*, *CLEC11A*); B cells (*CD79A*, *MS4A1*, *IGHM*, *CD19*); NKT cells (*NKG7*, *GZMK*, *GZMA*, *GZMB*, *CD3D*, *CD3E*, *CD3G*, *TRAC*); endothelial cells (*VWF*, *CAV1*, *CLDN5*, *EGFL7*, *PECAM1*) (Fig. 1C). Among all the cells detected in this study, T cells and myeloid cells were the most abundant, followed by CAFs and OB (osteoblast) cells (Fig. 1D). OB cells were primarily distributed in MLN and PT, whereas T cells and NKT cells were mainly found in LN (Fig. 1E).

### Transcriptional characteristics of osteoblast cells during the progression of osteosarcoma

**Fig. 2:**
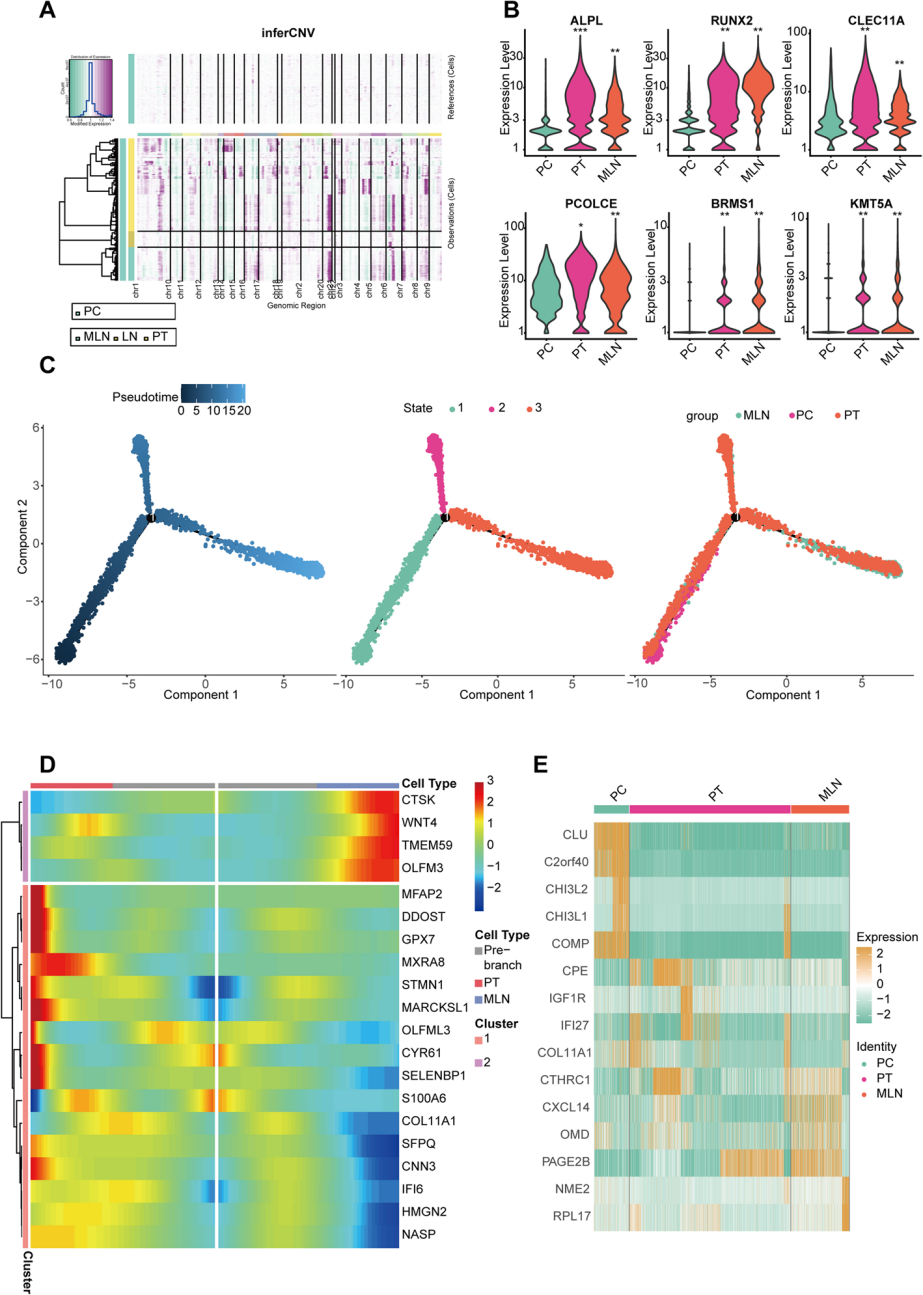
Transcriptional characteristics of osteoblast cells during the progression of osteosarcoma. **A** The results of CNV indicated that the copy number variation of OB cells in PT tissue was significantly more dynamic than in PC tissue. **B** The expression of the marker genes of osteoblastic cells and three genes associated with metastasis among PC, PT, and MLN. **C** The pseudotime trajectory during tumor progression. **D** The heat map of differential expression genes in different cell types of the pseudotime trajectory. **E** The heatmap of differential expression gene in OB cells across different groups (*p < 0.05, **p < 0.01, ***p < 0.001)

To identify the clonal structure and cell origin of malignant cells, we used the inferCNV algorithm to analyze CNVs and clonality in OB cells from MLN, LN, and PT samples. We chose OB cells from the PC sample as the reference, as PC osteoblasts were considered benign. The results showed that OB cells in PT and MLN samples exhibited increased gene copy numbers on most chromosomes except for chromosomes 10, 13, and 21, indicating that OB cells in PT and MLN were malignant osteosarcoma cells (OS cells). In contrast, OB cells in LN had similar copy numbers to reference cells, suggesting they were benign (Fig. 2A). Therefore, OB cells in PT and MLN were identified as malignant tumor cells, that is osteosarcoma cells (OS cells). Subsequently, we examined the expression abundance of osteoblast markers in PC, PT, and MLN. The results revealed low expression of ALPL, RUNX2, and CLEC11A in PC, while highly expressed in PT and MLN (all *p* < 0.05, Wilcoxon test), which suggested abnormal activation and malignant transformation of OB cells during tumor progression (Fig. 2B). Furthermore, we compared the expression of three genes that have been demonstrated in other studies to be associated with lung metastasis in OS [13–15]. The results indicate high expression of these genes in MLN and PT (all *p* < 0.05, Wilcoxon test), suggesting that these genes may also be involved in the LN metastasis (Fig. 2B). The results of the pseudotime trajectory demonstrated that PC cells were predominantly at the start of differentiation, MLN cells at the end, and PT cells distributed throughout various stages. We also performed pseudotime analysis for patient 7, who was simultaneously collected for PC, PT, and MLN. It can be seen that the results for patient 7 are similar to those of the pooled sample. This suggested, during the process of LN metastasis, OB cells may follow a differentiation path from PC → PT → MLN (state3) or from PC → PT (state2) (Fig. 2C, Additional file 5: Fig. S1). Many genes showed differential expression during the transition of states, indicating that these genes may mediate the pathogenesis and LN metastasis of OS (Fig. 2D). Additionally, the top 5 differentially expressed genes in various tissues have been presented. These genes may be involved in the pathogenesis and metastasis of OS and warrant further investigation (Fig. 2E). Besides, the functions of all differential genes among the three groups are illustrated in Fig. S2A (Additional file 6).

### ETS2 mediates LN metastasis through IBSP by bioinformatics

**Fig. 3:**
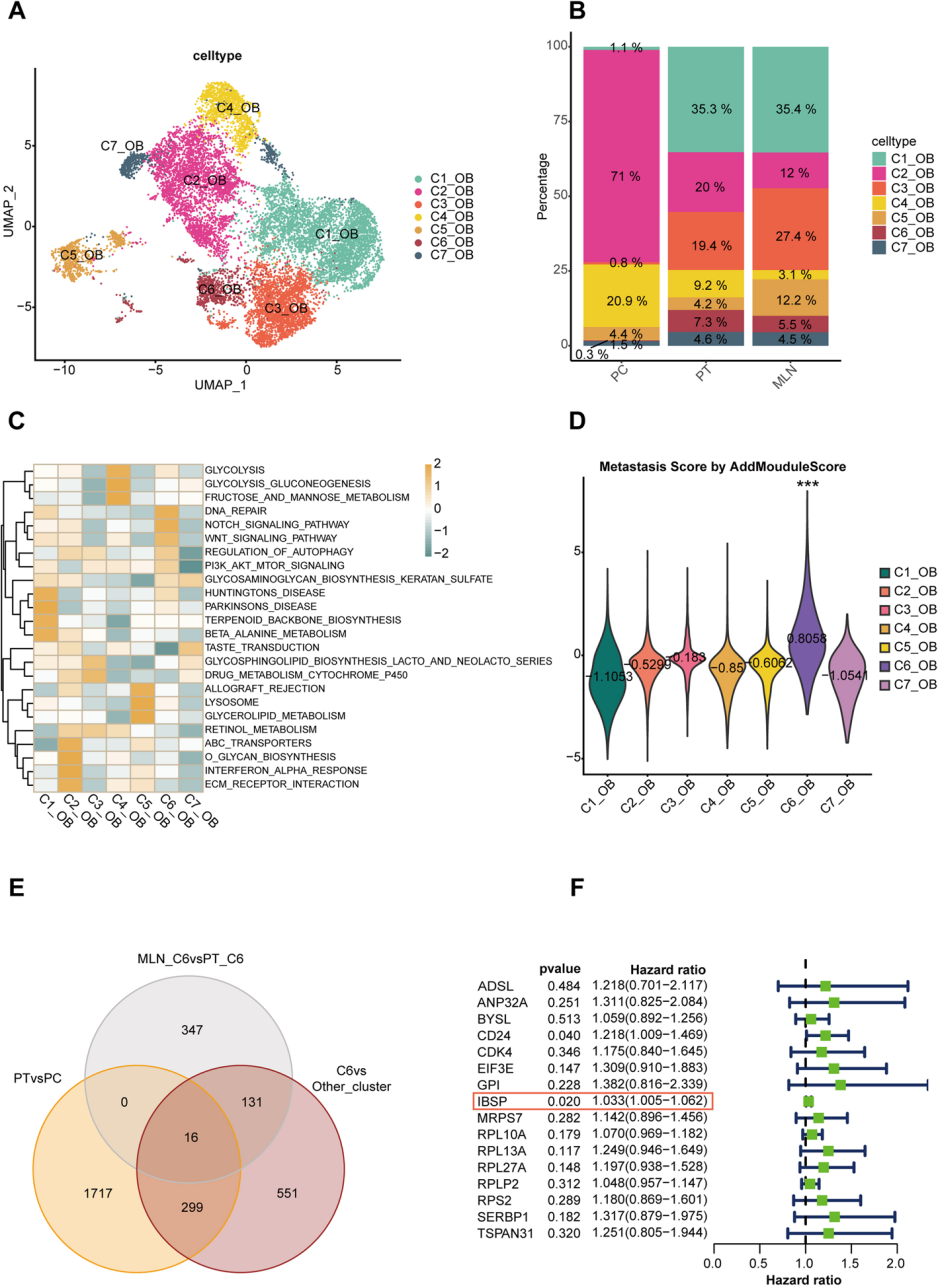
Osteoblast subtype classification. **A** OB were divided into seven distinct subgroups. **B** The composition of OB subpopulations between the different groups. **C** GSVA analysis showed that C6 clusters were significantly enriched in pathways related to DNA repair, NOTCH signaling pathway, and WNT signaling pathway. **D** Metastasis scores were significantly higher in C6 cluster cells by AddMouduleScore (***p < 0.001)

In order to further explore the mechanism of metastasis, we subdivided OB cells into 7 distinct subgroups (Fig. 3A). Meanwhile, we examined the composition of OB cells in samples from different sources and found significant heterogeneity among them (Fig. 3B). Figure S2B (Additional file 6) showed the top five most significantly differentially expressed genes in different cell subgroups. To elucidate the functions of different subgroups of OB cells, we performed GSVA analysis on the differentially expressed genes of each subgroup. The results revealed that the differentially expressed genes in the C6 cluster were significantly enriched in pathways related to metastasis such as DNA_REPAIR, NOTCH_SIGNALING_PATHWAY, WNT_SIGNALING_PATHWAY, and PI3K_AKT_MTOR_SIGNALING. Therefore, cells of the C6 cluster were considered the most likely subgroup executing the function of migration and invasion (Fig. 3C). To further confirm the association of the C6 subgroup with OS metastasis, we compiled genes previously reported to be related to metastasis in OS and scored each subgroup using these genes using AddModuleScore. The results indicated that the C6 subgroup had the highest score, suggesting that the C6 subgroup was most closely related to metastasis (Fig. 3D). Similar results were also applicable when using AUCell (Additional file 6: Fig. S2C). To identify hub genes in the C6 cluster, we intersected the following three gene sets: genes highly expressed in C6 of MLN compared to C6 of PT (MLN_C6 vs PT_C6), genes highly expressed in C6 compared to other subgroups in MLN (C6 vs other_clusters), and genes highly expressed in OB of PT compared to OB of PC (PT vs PC). There were 16 hub genes that were obtained (Fig. 3E). Next, we performed a survival analysis of these candidate genes in the TARGET database and found that only two genes (IBSP and CD24) possessed significant prognostic value (Fig. 3F). The IBSP gene encodes bone sialoprotein, which is involved in cell adhesion and migration and may be related to tumor metastasis. Therefore, IBSP is considered to be a key gene in subgroup C6 that is involved in metastasis. Upon consulting the PROMO database, it was discovered that ETS2 might be a transcription factor regulating IBSP (Additional file 6: Fig. S2D). Correlation analysis indicated that ETS2 has a regulatory effect on IBSP (Additional file 6: Fig. S2E). Therefore, we consider ETS2 as a potential transcription factor regulating IBSP.

### Validation of ETS2 to promote osteosarcoma cell metastasis via IBSP in vitro and in vivo

**Fig. 4:**
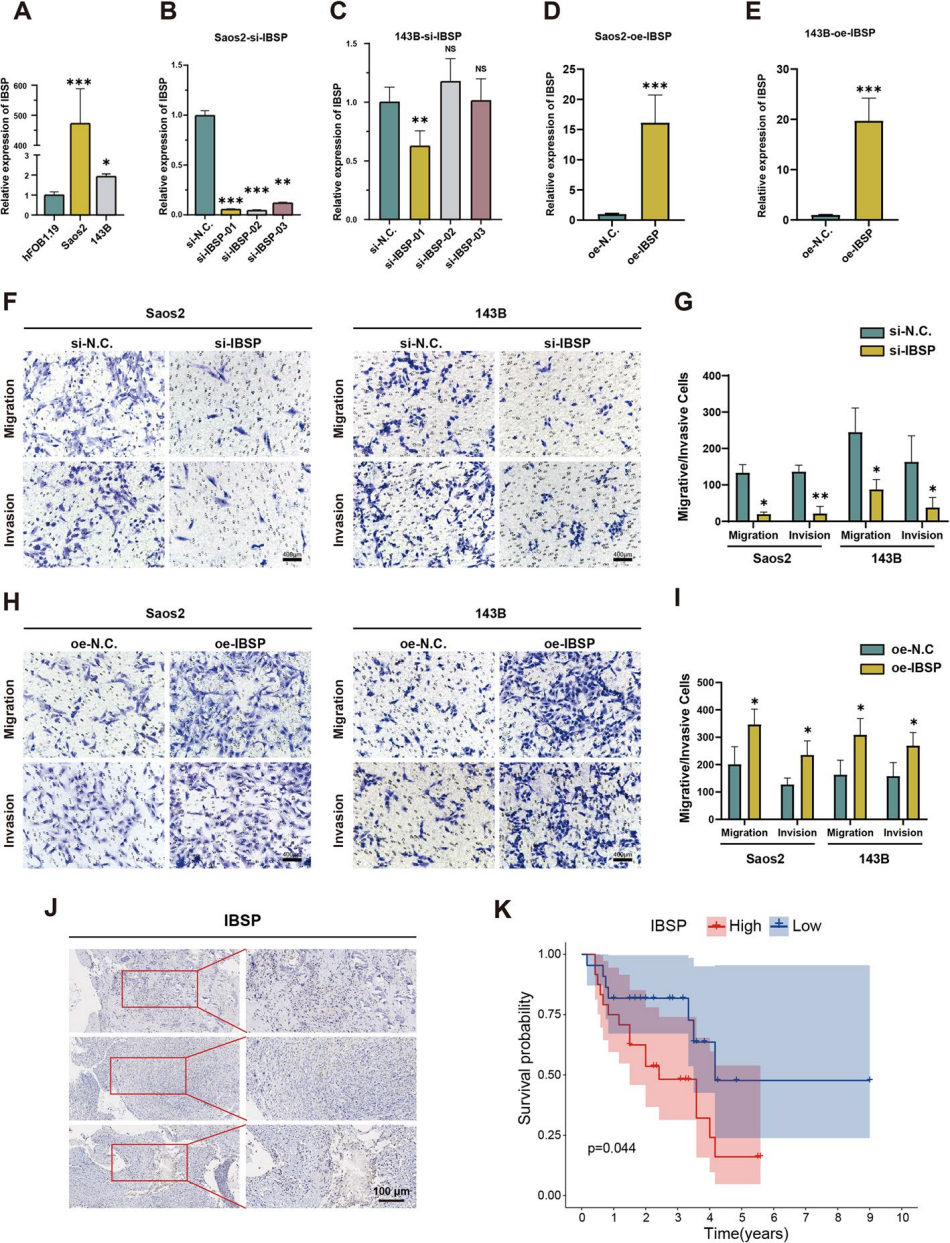
Validation of the effect of IBSP on osteosarcoma progression in vitro. **A** The expression level of IBSP was higher in Saos2 and 143B compared to hFOB1.19. **B**–**E** Functional assays for silencing and overexpression of IBSP. **F**, **G** Transwell migration and invasion assays for silencing of IBSP. **H**, **I** Transwell migration and invasion assay for overexpression of IBSP. **J** Immunohistochemical staining of osteosarcoma tissues confirms IBSP expression. **K** The Kaplan-Meier curve for IBSP (*p < 0.05, **p < 0.01, ***p < 0.001)

To investigate the impact of IBSP on tumor progression, we first compared the expression levels of IBSP between osteosarcoma cell lines (143B, Saos2) and normal osteoblast cells (hFOB1.19) using RT-qPCR assay. We found that the expression of IBSP was higher in Saos2 and 143B compared to hFOB1.19 (Fig. 4A). Subsequently, we performed silence and overexpression functional experiments of IBSP and ETS2 in the osteosarcoma cell lines. In the initial step, we designed siRNAs overexpression lentiviral vector targeting IBSP and conducted preliminary transfection in Saos2 and 143B cells. The results showed that si-IBSP 01 exhibited the best knockdown efficiency (Fig. 4B and C), and the overexpression lentiviral vector possesses prominent overexpressed efficiency (Fig. 4D and E). Thus, si-IBSP 01 and the overexpression lentiviral vector of IBSP were used for further experiments. Transwell migration and invasion assays showed that IBSP knockdown significantly reduced the migration and invasion ability of Saos2 and 143Bcells (Fig. 4F and G). On the contrary, overexpressing IBSP significantly increased the migration and invasion ability of these two cells (Fig. 4H and I). Subsequently, we conducted immunohistochemical staining, confirming the expression of IBSP in osteosarcoma tissues (Fig. 4J). Kaplan-Meier curves demonstrated that high expression of IBSP was associated with poor prognosis in patients (*p* < 0.05) (Fig. 4K), further supporting the involvement of IBSP in tumorigenesis and progression. In addition, EdU staining demonstrated a significant decrease in cell proliferation and EdU incorporation in siRNA cells compared to the control group (Additional file 7: Fig. S3A–C).

**Fig. 5:**
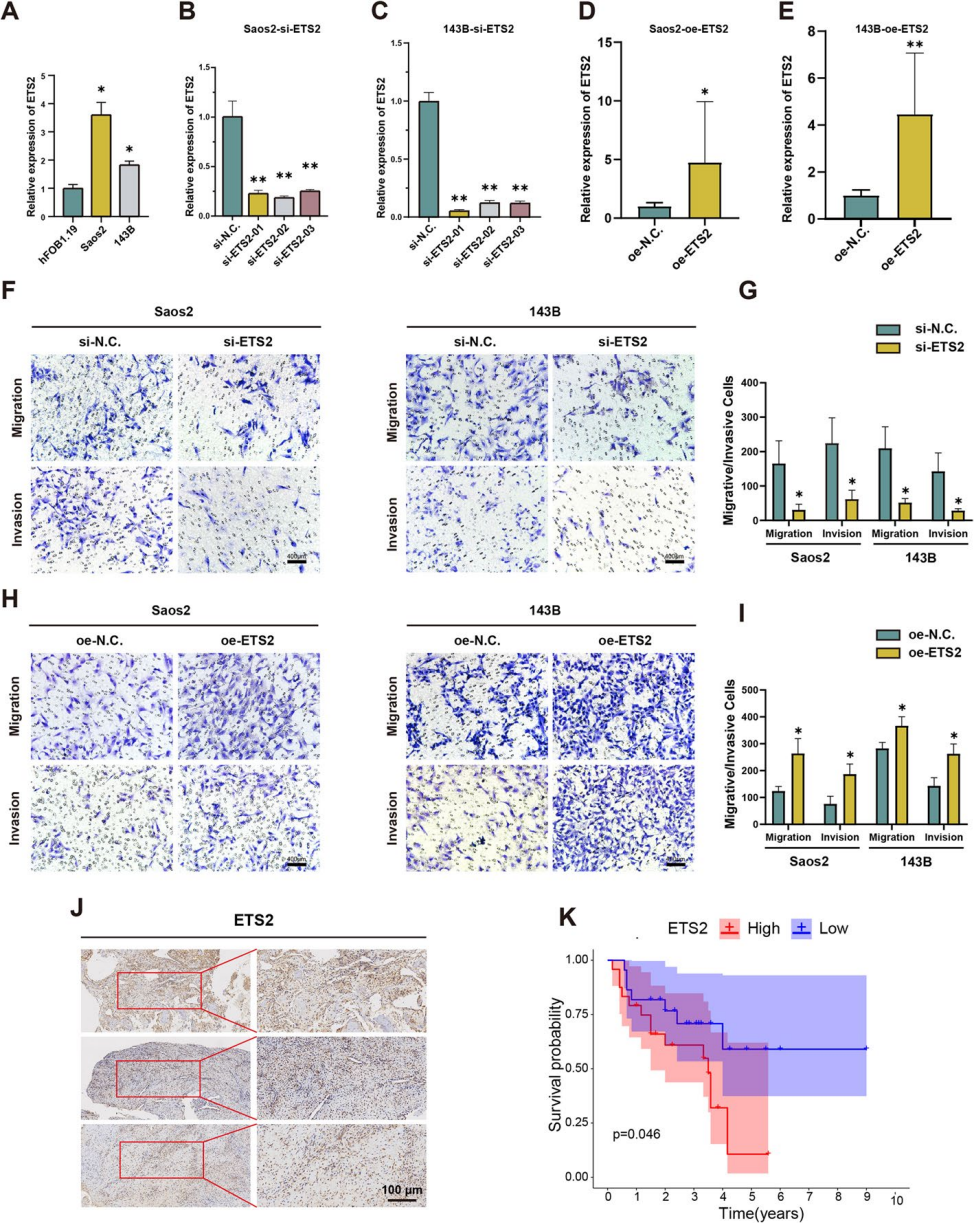
Validation of the effect of ETS2 on osteosarcoma progression in vitro. **A** The expression level of ETS2 was higher in Saos2 and 143B compared to hFOB1.19. **B**–**E** Functional assays for silencing and overexpression of ETS2. **F**, **G** Transwell migration and invasion assays for silencing of ETS2. **H**, **I** Transwell migration and invasion assay for overexpression of IBSP. **J** Immunohistochemical staining of osteosarcoma tissues confirms ETS2 expression. **K** The Kaplan-Meier curve for ETS2 (*p < 0.05, **p < 0.01, ***p < 0.001)

Similarly, ETS2 was also highly expressed in osteosarcoma cell lines (Fig. 5A). The si-ETS2 01, the best knockdown efficiency, and the overexpression plasmid of ETS2 show remarkable overexpressed efficiency, which were selected for further study (Fig. 5B–E). Transwell migration and invasion assays showed that ETS2 knockdown significantly reduced the migration and invasion ability of Saos2 and 143B (Fig. 5F and G), while the trend was elevated when ETS2 was overexpressed (Fig. 5H and I). What’s more, the result of immunohistochemical staining released that high expression of ETS2 led to poor prognosis (*p* < 0.05) (Fig. 5J and K). EdU assay showed that si-ETS2 would result in a significant decrease in cell proliferation (Additional file 7: Fig. S3D–F).

**Fig. 6:**
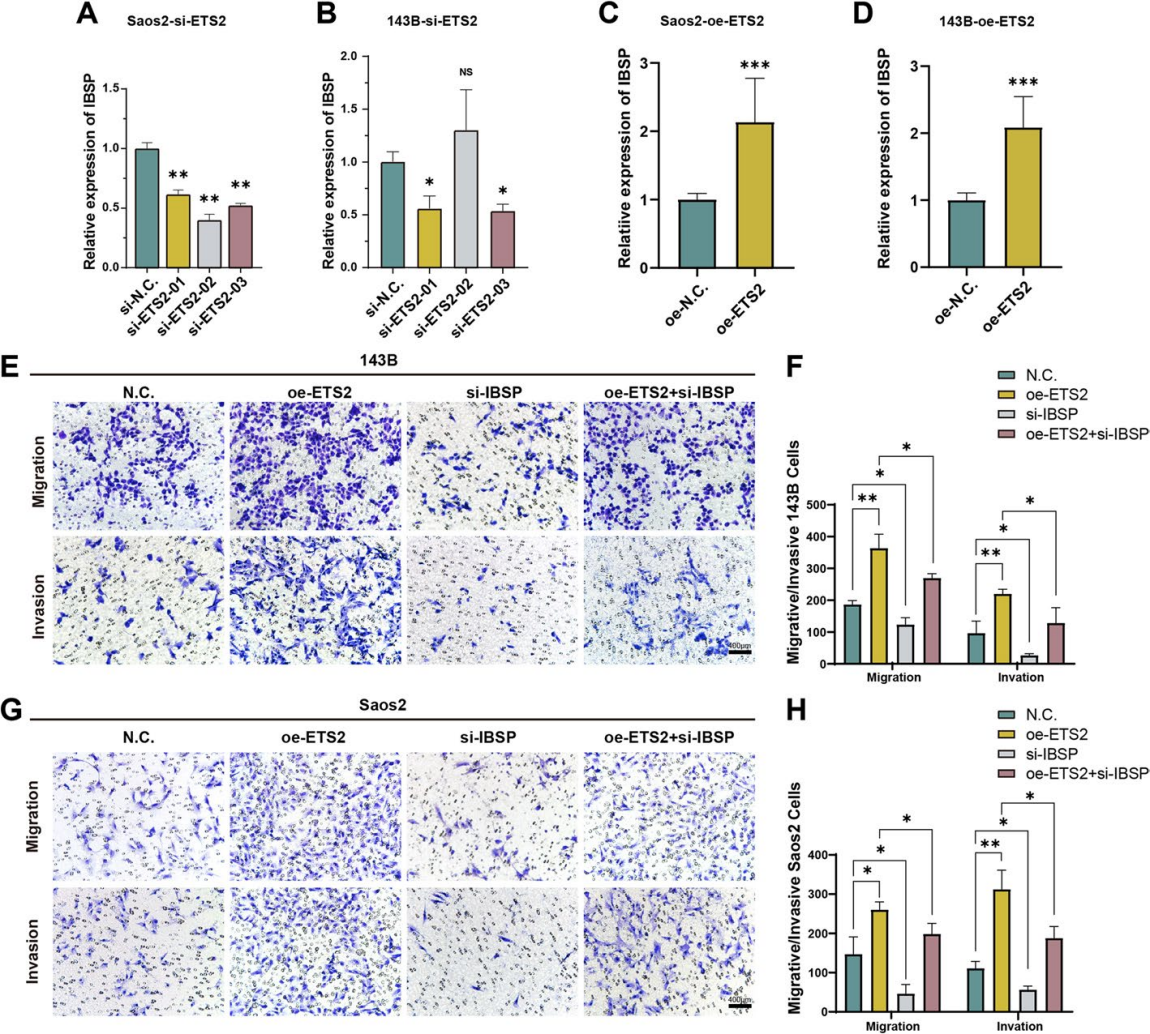
In vitro experiments validate the effect of IBSP combined with ETS2 on osteosarcoma. **A**, **B** Histogram of IBSP expression upon silencing of ETS2. **C**, **D** Histogram of IBSP expression upon overexpression of ETS2. **E**-**H** Migration and invasion assays for NC, oe-ETS2, si-IBSP, and oe-ETS2 + si-IBSP (*p < 0.05, **p < 0.01, ***p < 0.001)

Furthermore, the expression of IBSP will be reduced or increased when ETS2 was silenced and overexpressed, which demonstrated that ETS2 could regulate the expression of IBSP (Fig. 6A–D). To further validate the regulatory relationship between ETS2 and IBSP, four groups (NC, oe-ETS2, si-IBSP, and oe-ETS2 + si-IBSP) were established separately using Saos2 and 143B cells for migration and invasion experiment. The results suggested that oe-ETS2 enhanced the migration and invasion and si-IBSP reduced migration and invasion. The ability of migratory invasion in the oe-ETS2 group was significantly reduced when IBSP was silenced, which demonstrated that ETS2 exerted its function via IBSP (Fig. 6E–H).

**Fig. 7:**
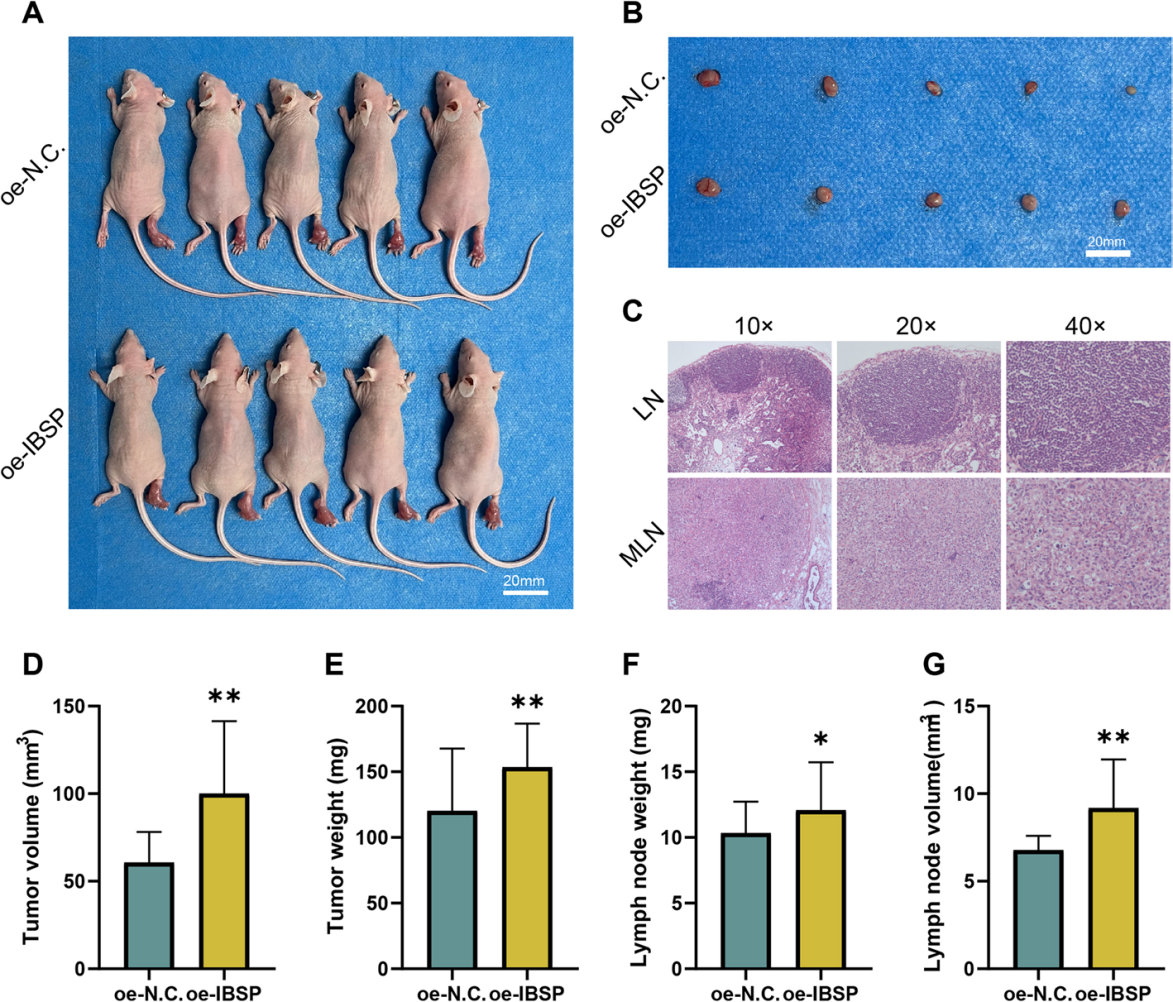
IBSP may promote osteosarcoma progression in vivo. **A** The gross view of whole-body of nude mice in NC and oe-IBSP groups. **B** The gross view of tumors in nude mice in the NC and oe-IBSP groups. **C** HE staining of MLN and LN in the groin of nude mice. **D**, **E** The bar graphs of tumor volume and weight in the NC and oe-IBSP groups. **F**, **G** The bar graphs of lymph node volume and weight in the NC and oe-IBSP groups (*p < 0.05, **p < 0.01, ***p < 0.001)

At the in vivo experimental level, oe-IBSP143B cells were injected into the right footpad of nude mice to establish the lymph node metastasis model. The results indicated that, compared to the NC group, the volume and weight of tumors in the oe-IBSP group significantly increased, suggesting that IBSP may promote the proliferation of osteosarcoma cells (Fig. 7A, D–E). Furthermore, the volume and weight of inguinal lymph nodes in the oe-IBSP group exhibited a notable increase (Fig. 7B, F–G). Subsequently, HE staining clarified that two cases in the NC group and four cases in the oe-IBSP group developed into metastasis in the inguinal lymph nodes, indicating that IBSP can significantly promote lymph node metastasis in vivo (Fig. 7C). In conclusion, these results suggested that the axis of ETS2/ IBSP might promote the progression of OS cells.

### Tumor microenvironment characteristics of MLN

We meticulously compared the differences in cell proportions between MLN and LN and found that T cells were significantly reduced in MLN, while CAFs and myeloid cells were notably increased (Fig. 1E and Additional file 8: Fig. S4A). Therefore, we will focus on the role of CAFs and myeloid cells in remodeling the LN.

**Fig. 8:**
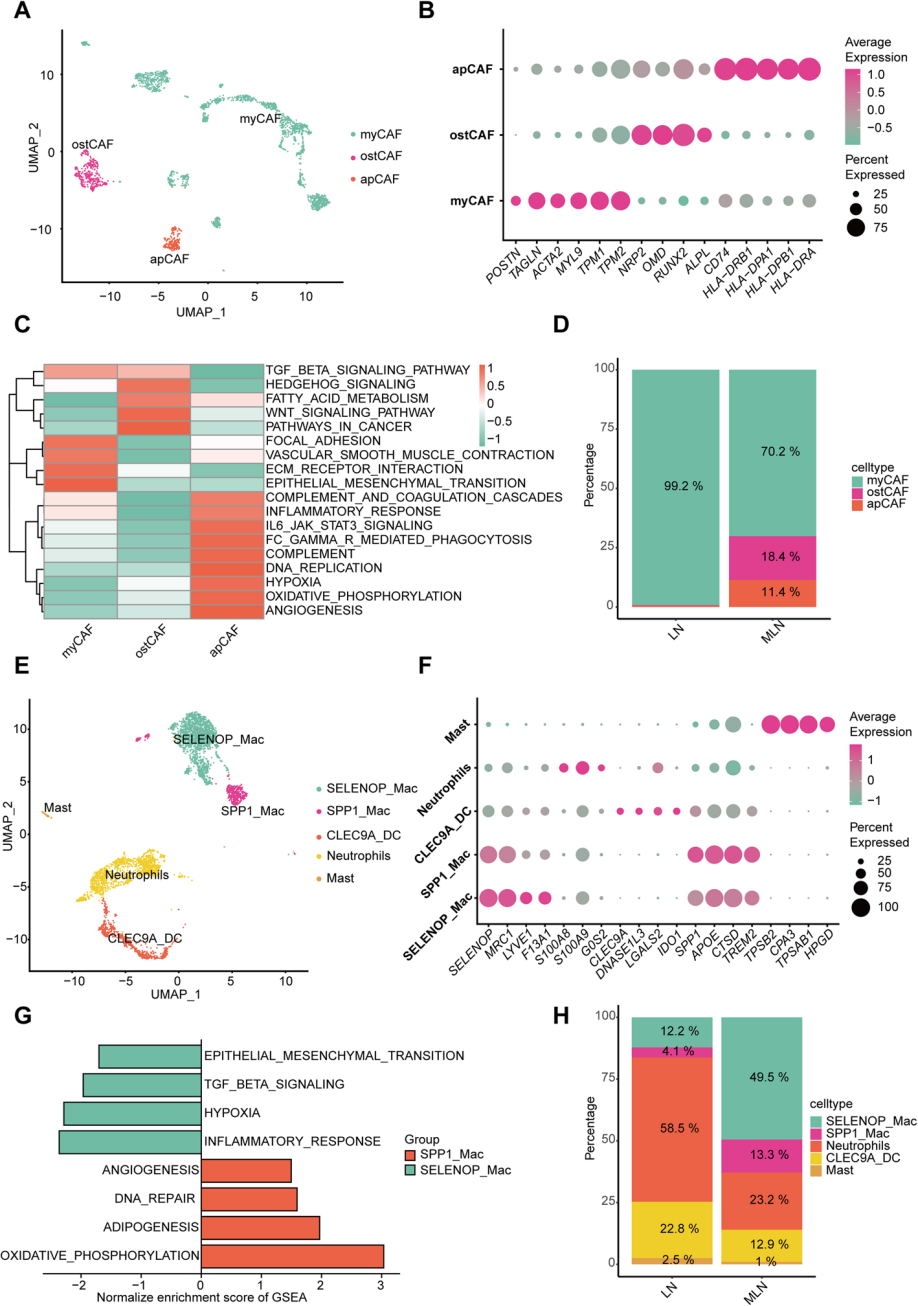
Tumor microenvironment characteristics of MLN. **A** CAFs in MLN and LN are defined as 3 clusters, namely osteoblast-like CAFs (OstCAF), myCAF, and apCAF. **B** The bubble map of marker genes for each cluster of CAFs. **C** The heatmap of GSVA gene enrichment analysis for each CAF cluster. **D** The stacked histogram showed that significant increase in the proportion of OstCAF and ApCAf and a decrease in myCAF in MLN. **E** The further cluster of myeloid cells in MLN and LN. **F** The bubble map of marker genes for each cluster of myeloid cells. **G** The bar chart of GSEA gene enrichment analysis for each myeloid cell cluster. **H** Stacked Histogram showed that significant decrease in the proportion of SELENOP Mac and SPP1 Mac

Firstly, based on marker gene expression, CAFs were further classified into three subgroups, namely osteoblast-like CAFs (osteogenic CAFs, OstCAFs), myCAFs, and apCAFs (Fig. 8A and B). MyCAFs expressed high levels of extracellular matrix and actomyosin-related proteins such as POSTN and TAGLN. GSVA analysis suggested a correlation with pathways like VASCULAR_SMOOTH_MUSCLE_CONTRACTION and ECM_RECEPTOR_INTERACTION (Fig. 8C). OstCAFs expressed numerous genes similar to OS cells (ALPL, RUNX2), with GSVA linking them to TGF_BETA_SIGNALING_PATHWAY, PATHWAYS_IN_CANCER, and WNT_SIGNALING_PATHWAY, highlighting their involvement in tumor progression (Fig. 8C). ApCAFs were characterized by high expression of antigen presentation molecules (HLA-DRA, HLA-DPA1, HLA-DPB1, HLA-DRB), and the pathway was associated with immune regulation (Fig. 8C). Compared to LN, MLN shows a significant increase in OstCAFs (Fig. 8D). CellphoneDB analysis reveals distinct interactions between OB cells and OstCAFs (Additional file 8: Fig. S4B and C). Additionally, genes associated with osteogenesis are notably overexpressed in MLN, while they are virtually unexpressed in LN (Additional file 8: Fig. S4D). In summary, these results suggested that tumor cells may create a favorable microenvironment for their own generation by OstCAFs.

Based on the markers’ gene expression, myeloid cells can be divided into five groups: SELENOP_Mac, SPP1_Mac, CLEC9A_DC, neutrophils, and mast cells (Fig. 8E and F). Macrophages are the primary functional subgroup of myeloid cells, warranting further investigation. It was found that SPP1_Mac is mainly associated with pathways such as angiogenesis, and SELENOP_Mac was principally related to epithelial-mesenchymal transition, indicating that both these macrophage groups potentially promote tumor progression (Fig. 8G). SPP1_Mac and SELENOP_Mac were significantly increased in MLN (Fig. 8H), and CellphoneDB indicates a strong interaction with OB cells (Additional file 8: Fig. S4B). This suggests that OB cells may promote their own development via SELENOP_Mac and SPP1_Mac.

### Spatial transcriptomic characteristics of MLN

**Fig. 9:**
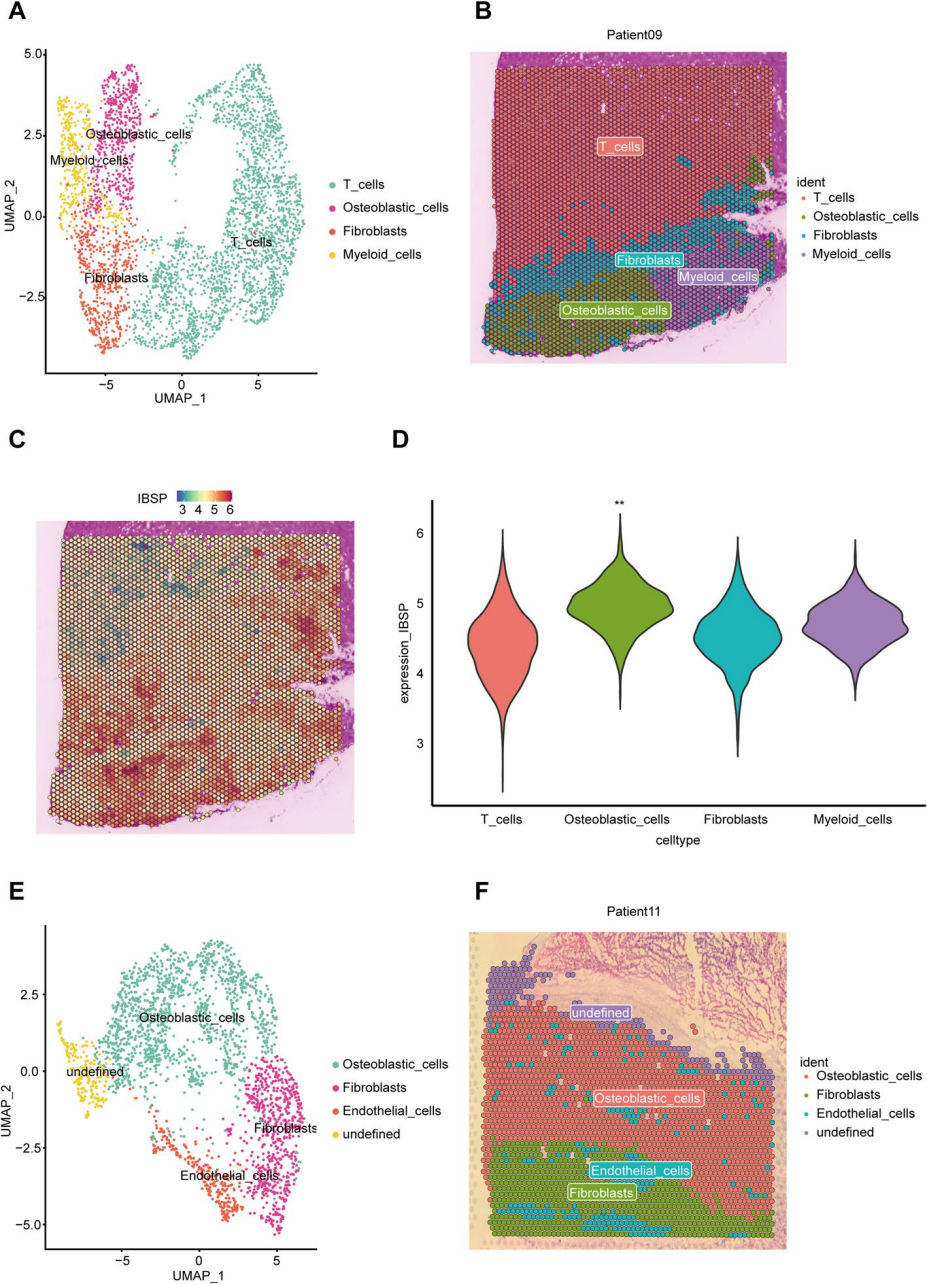
Spatial transcriptomic characteristics of MLN. **A** The UMAP plot of spatial transcriptomic sequencing in patient 9. **B** The spatial locations of each subgroup in patient 9. **C** The spatial locations of IBSP expression expressed in the spatial transcriptomic data of patient 9. **D** The violin plot of IBSP expression of each cell subgroup in the spatial transcriptomic data of patient 9. **E** The UMAP plot of spatial transcriptomic sequencing in patient 11. **F** The spatial locations of each subgroup in patient 11

Subsequently, spatial transcriptomic analysis was performed on two metastatic lymph node samples. After quality control, dimensionality reduction, and clustering, the spots from patient 09 were divided into five subgroups. Deconvolution algorithms determined that subgroups 0 and 1 were T cells, subgroup 4 was CAFs and subgroup 4 was myeloid cells (Additional file 9: Fig. S5A and B). Subgroup 2, in conjunction with the AddModuleScore algorithm, was identified as OB cells (Additional file 9: Fig. S5C). In terms of spatial distribution, OB cells were located adjacent to CAFs and myeloid cells, which further confirms the interaction between OB cells and these two cell types. Interestingly, CAFs act as a barrier, isolating T cells and thereby inhibiting the cytotoxic effect of T cells on OB cells (Fig. 9A). Besides, IBSP was highly expressed in OB cells, which corroborates the notion once again that high expression of IBSP may lead to LN metastasis (Fig. 9B and C). For patient 11, the spots were divided into five subgroups. Subgroups 0 and 2 were identified as OB cells, subgroup 1 as CAFs, and subgroup 3 as endothelial cells (Additional file 9: Fig. S5D–F). Although there were no T cells and myeloid cells in the MLN of patient 11, CAFs were still observed adjacent to OB, further corroborating the interactive influence of CAFs on OB. Additionally, endothelial cells were interspersed within the CAFs and OB, providing nutritional support for tumor growth (Fig. 9D).

## Discussion

**Fig. 10:**
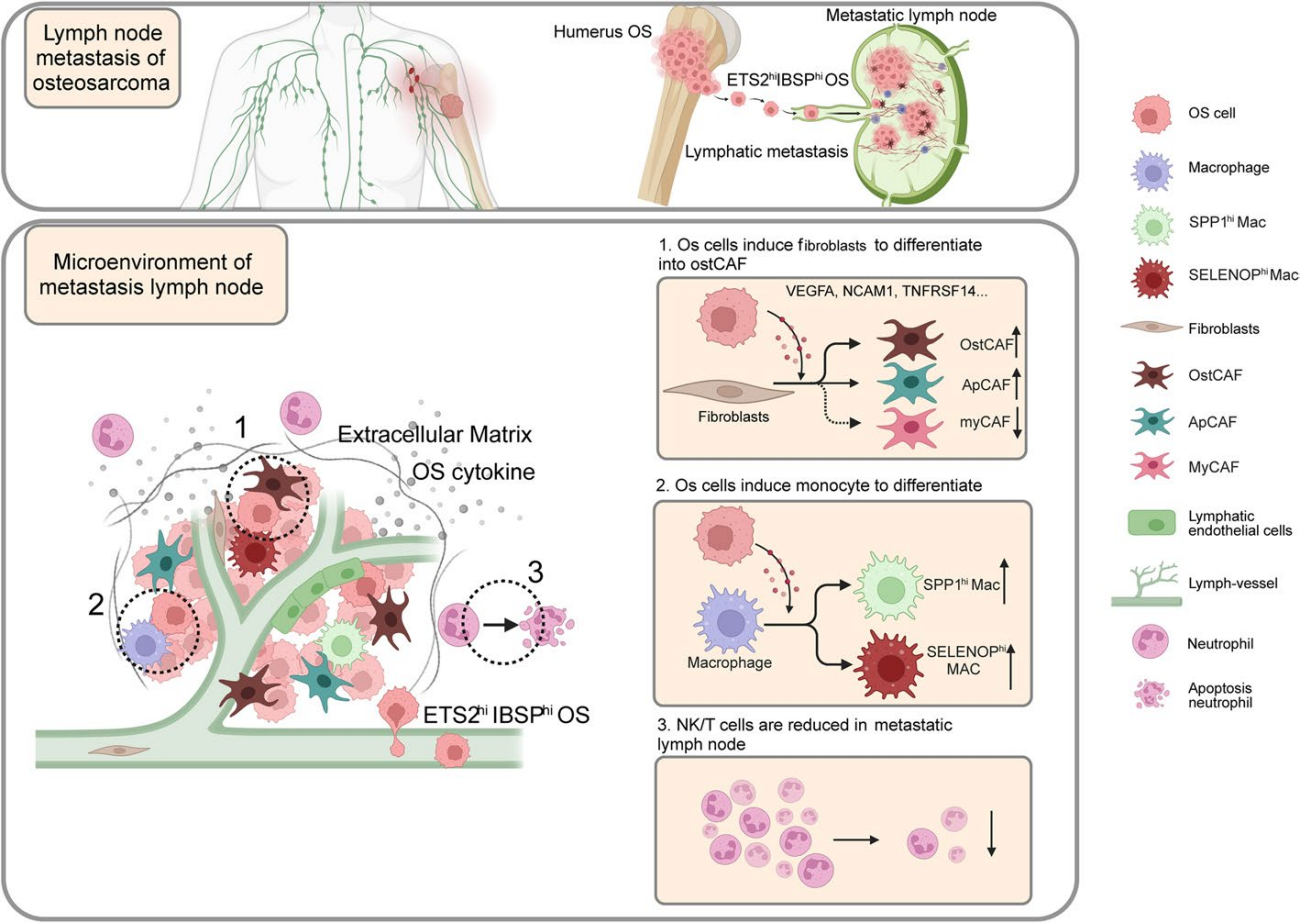
Mechanism diagram of the present study

Osteosarcoma is a highly malignant tumor with a poor prognosis and patients will suffer from lower survival rates when LN metastasis occurs. Most studies have focused on hematogenous metastasis of osteosarcoma, but few research has been done on the specific mechanisms of LN metastasis. Meanwhile, there is nearly a gap in the research of LN microenvironment remodeling by tumor cells. Thus, for the first time, we precisely dissected the molecular mechanism of LN metastasis in osteosarcoma at the single-cell level. We identified metastasis-associated subpopulations of OS cells and validated the regulatory role of the ETS2/IBSP signaling axis in LN metastasis via in vitro and in vivo experiments. Besides, by combining scRNA sequencing and corresponding spatial transcriptome data, OS cells were found to improve adaptability via interacting with myeloid cells (mainly macrophages) and CAFs (Fig. 10).

ScRNA sequencing technique is becoming more widely used in the study of tumor metastasis. Peizhan Chen et al., for example, explored the evolution of malignant epithelial cells in paracancerous tissue, primary gallbladder cancer, and MLN via scRNA sequencing [16]. Professor Xu and his colleagues, for instance, used scRNA sequencing to identify a tumor cell subset with high expression of CXCL1, which was confirmed to promote LN metastasis in breast cancer [17]. Researchers from another study also determined a subset related to LN metastasis in head and neck squamous cell carcinoma and characterized the changes of molecules and pathways in the metastasis process by scRNA sequencing [18]. In our previous study, we performed scRNA sequencing for primary cancer from six osteosarcoma patients. However, due to technical limitations at the time, it was not possible to digest hard bone, resulting in the lack of paracancer controls [12]. In the present study, eight primary osteosarcoma lesions, four paracancerous, four non-MLN, and two MLN were included for scRNA sequencing. The significant CNV variation of OB cells indicated that it was the predominant malignant cells in OS. In addition, the transcriptome of those three kinds of issues was compared, and pseudotime analyses were performed. The result indicated that OB cells were continuously activated during the evolution. We also identified some genes associated with LN metastasis, which might provide a theoretical basis for further studies.

The theory of tumor heterogeneity held that the same cellular component in tumor tissue also has different biological functions. For instance, Yan Zhou et al. performed scRNA sequencing for primary osteosarcoma, recurrent osteosarcoma, and lung metastasis lesions, and further identified OB cells into 6 subgroups which exerted different functions [19]. In the present study, we divided OB cells into seven subpopulations and found that cluster 6 (C6) might play a key role in LN metastasis by GSVA analysis and metastasis score. Subsequently, integrin-binding sialoprotein (IBSP), which was highly expressed in C6, was identified as a critical gene for LN metastasis. Subsequently, the result of the PROMO database shows that E-twenty six 2 (ETS2) was the transcription factor of IBSP.

IBSP is located on chromosome 4, region 2, band 2, sub-band 1 region, and its protein is an acidic glycoprotein with a molecular mass of approximately 70 kDa [20]. IBSP is highly expressed in a variety of malignant tumors, including breast cancer, prostate cancer, lung cancer, and esophageal squamous cell carcinoma, which is considered to be a promoting factor for tumor migration and invasion [21]. However, the role of IBSP in mediating invasion and metastasis in osteosarcoma is still unclear. ETS2 is a transcription factor that is involved in cell proliferation and migration and has been shown to be closely related to tumor development, angiogenesis, and metastasis. Research showed that knockdown of ETS2 inhibited lung metastasis in OS [22], strongly suggesting that ETS2 is associated with metastasis. However, could ETS2 regulate LN metastasis in OS, and whether it regulates the expression of IBSP? None of these questions are clear. In our study, the role of the ETS2/IBSP signaling axis in LN metastasis was initially investigated by scRNA sequencing data and bioinformatics analysis. Besides, in vitro experiments showed that the expression of ETS2 and IBSP was both higher in OS cells. Silencing these two genes will result in weakened proliferation, migration, and invasion of the tumor, whereas overexpression shows the opposite trend. In vivo experiments also confirmed that overexpressing IBSP promoted tumor growth and lymph node metastasis in mice. In brief, we identified and verified the key role of ETS2/IBSP, a new signaling axis, in LN metastasis in osteosarcoma, which could provide new targets and direction to block metastasis.

Another important aspect of metastasis is to investigate how tumor cells remodel the microenvironment of metastatic focus [23]. As we know, there are a large number of immune cells in normal LN, such as B cells, T cells, and NK cells, which are a barrier against the spread of bacteria and tumor cells [24]. However, this normal environment will be broken by the tumor cells, which will rebuild their “residence” through cellular interactions after they are planted [9]. T cells are essential for killing tumor cells in LN. Research showed that tumor cells can directly affect T cells, which would lead to immune escape [9]. Our data showed a significant reduction of T cells in MLN, suggesting that tumor cells might decrease the number of T to create an environment suitable for their own survival. Tumor-associated myeloid cells are characterized by their high heterogeneity and plasticity, with tumor-associated macrophages (TAMs) being the primary functional subgroup [25]. TAMs were initially thought to exert anti-tumor effects by killing tumor cells or presenting tumor antigens to induce an immune response, thereby clearing the tumor [26]. However, further research has revealed that the main role of TAMs is to promote tumorigenesis and immune suppression [27]. TAMs are classified into two subgroups by recent scRNA seq techniques: SPP1^+^ Mac and SELENOP^+^ Mac. It is believed that SPP1^+^ Mac promotes tumor growth through mechanisms such as immune suppression, epithelial-mesenchymal transition, angiogenesis, and remodeling of the matrix of CAFs [28, 29]. Research indicates that SELENOP+ Mac can promote anti-tumor immunity by highly expressing FOLR2, IL32, CD3D, and LTC4S and are therefore considered to have anti-tumor effects [30]. However, another study suggested that SELENOP+ Mac was similar to M2-type macrophages and had a role in promoting tumor growth [31]. In the present study, both SPP1^+^ Mac and SELENOP^+^ Mac in MLN were found to promote tumor cell growth and were significantly increased in MLN. In terms of spatial distribution, they were located near OB cells and, in conjunction with CAFs, form a barrier that blocks T cells and provide a favorable microenvironment for tumor growth. Besides immune cells, tumor cells can also interact with stromal cells. Fibroblasts (CAFs) are the most important stromal cells in LN. Under physiological conditions, CAFs can produce chemokines to enhance the immune capacity of LN [32]. However, CAFs can also be reprogrammed by tumor cells for immune escape [33]. CAFs often envelop tumor cells, forming a barrier that isolates immune cells. CAFs can also secrete numerous cytokines, such as ACTA2, FBLN1, TAGLN, etc., remodeling the tumor extracellular matrix, thereby leading to tumor progression. Furthermore, CAFs can directly promote tumor growth by secreting various proteins. In the present study, we found that the proportion of OstCAF, a novel subgroup that promotes tumor progression, was significantly higher in MLN. Besides, spatial transcriptome data further confirmed that tumor cells were bordered by CAFs, and the T cells were isolated. Therefore, we believe that CAFs play a crucial role in the survival of tumor cells within the lymph nodes.

Although the present study provides some novel and valuable data and results, there are still many limitations. First, the sample size of LN is relatively small, and more sequencing samples need to be included to confirm and extend our findings. Secondly, the spatial transcriptome data of osteosarcoma tissues are still missing in this study because of osteogenic components, which are difficult to be permeabilize in experiments. Finally, the conclusions about LN microenvironment remodeling in this study were deduced from sequencing data, which need to be further validated by more experiments.

Although the present study provides some novel and valuable data and results, there are still many limitations. First, the sample size of LN is relatively small, and more sequencing samples need to be included to confirm and extend our findings. Secondly, the spatial transcriptome data of osteosarcoma tissues are still missing in this study because of osteogenic components, which are difficult to be permeabilize in experiments. Finally, the conclusions about LN microenvironment remodeling in this study were deduced from sequencing data, which need to be further validated by more experiments.

## Conclusions

In conclusion, this study combined scRNA sequencing and spatial transcriptome technology to identify a subpopulation of OS cells that perform metastatic functions. Moreover, we discovered and validated the role of a new signaling axis, ETS2/IBSP, in LN metastasis of osteosarcoma.

### Supplementary Information

**Additional file 1: Table S1.** Clinical characteristics of patients in the present study.

**Additional file 2: Table S2.** Information Sheet on Genes Associated with Osteosarcoma Lung Metastasis Score.

**Additional file 3: Table S3.** The siRNA sequences.

**Additional file 4: Table S4.** Sequence of the primer of IBSP and ETS2.

**Additional file 5: Figure S1.** The pseudotime analysis for Patient 7

**Additional file 6: Figure S2.** (A) The heatmap of GSVA analysis of OB from different tissue sources (B) The heat map of differential genes in clusters of OB cells. (C) Metastasis scores significantly higher in C6 cluster cells by AUCell. (D) Sequences predicted by the promo database for possible binding of IBSP with ETS2. (E) Correlation analysis of IBSP and ETS2 expression in OB cells. (***p < 0.001)

**Additional file 7: Figure S3.** (A-C) Significantly reduced admixture of EdU in si-IBSP cells compared to controls in two osteosarcoma cell lines. (D-F) Significantly reduced admixture of EdU in si-ETS2 cells compared to controls in two osteosarcoma cell lines.

**Additional file 8: Figure S4.** (A) The UMAP plot of cell clusters categorized by LN and MLN. (B) The heat map of the strength of cellular interaction relationships between myeloid cells and OBs. (C) The bubble plot of the ligand-receptor relationship between myeloid cells and OBs. (D) The bubble plot of OB markers expression in LN and MLN.

**Additional file 9: Figure S5.** (A) The spatial location of initial subgroups of patient 9. (B) Inverse convolution was used to define subgroups 0 and 1 as T cells, subgroup 3 as fibroblasts, and subgroup 4 as myeloid cells in patient 9. (C) Subgroup 3 was identified as osteoblastic cells by AddModuleScore. (D) The spatial location of initial subgroups of patient 9. (E) Inverse convolution was used to define subgroups 0 and 2 as osteoblastic cells and subgroup 1 as fibroblasts in patient 9. (F) Subgroup 3 was identified as endothelial cells by AddModuleScore.

## Data Availability

The raw data and processed gene expression data of single-cell RNA and spatial transcriptome sequencing are available at the Genome Sequence Archive (GSA) of the National Genomics Data Center (access number: HRA007229 and PRJCA024817) and Gene Expression Omnibus (GEO) database under accession code GSE237070 and GSE162454. (GSE162454:Liu Y, Feng W, Dai Y, et al. Single-cell RNA sequencing of human osteosarcoma https://www.ncbi.nlm.nih.gov/geo/query/acc.cgi?acc=gse162454 (2021)). The remaining data are available within the Article, Supplementary Information. Source data are provided in this paper. This paper does not report the original code. Any additional information required to reanalyze the data reported in this paper is available from the lead contact upon request.

## Abbreviations

CAF: Cancer-associated fibroblast
CNV: Copy number variation
DPBS: Dulbecco’s phosphate-buffered saline
ETS2: ETS Proto-Oncogene 2
HE: Hematoxylin and eosin
IBSP: Integrin binding sialoprotein
LEC: Lymphatic endothelial cell
LN: Lymph node
MLN: Metastatic lymph node
OB: Osteoblast
OS: Osteosarcoma
PBS: Phosphate-buffered saline
PC: Paracancerous tissue
PT: Primary tumor
scRNA: Single-cell RNA
