# Supplementary material for: Single-cell and spatial transcriptomics reveal metastasis mechanism and microenvironment remodeling of lymph node in osteosarcoma
Source: BMC Med. 2024 May 17;22:200. doi: 10.1186/s12916-024-03319-w (PMC11100118; doi:10.1186/s12916-024-03319-w)
Supplement: Supplementary file 1 — Additional file 1: Table S1. Clinical characteristics of patients in the present study. [file 12916_2024_3319_MOESM1_ESM.docx]

| ID | Gender | Age | Location | Primary tumor | Paracancerous | Lymph nodes |
| --- | --- | --- | --- | --- | --- | --- |
| Patient 1 | Male | 16Y | Distal femur | PT1 | PC1 | NA |
| Patient 2 | Female | 19Y | Distal femur | PT2 | NA | NA |
| Patient 3 | Female | 45Y | Middle femur | PT3 | PC2 | NA |
| Patient 4 | Male | 19Y | Middle femur | PT4 | NA | NA |
| Patient 5 | Male | 14Y | Proximal tibia | PT5 | NA | NA |
| Patient 6 | Male | 13Y | Distal femur | PT6 | NA | NA |
| Patient 7 | Female | 34Y | Proximal humerus | PT7 | PC3 | MLN |
| Patient 8 | Female | 14Y | Proximal tibia | PT8 | PC4 | LN |
| Patient 9 | Male | 16Y | Distal femur | NA | NA | LN/MLN |
| Patient 10 | Female | 40Y | Proximal Humerus | NA | NA | LN |
| Patient 11 | Male | 14Y | Distal femur | NA | NA | LN |

Table S1 Clinical characteristics of patients in the present study.
