# Supplementary material for: Single-cell and spatial transcriptomics reveal metastasis mechanism and microenvironment remodeling of lymph node in osteosarcoma
Source: BMC Med. 2024 May 17;22:200. doi: 10.1186/s12916-024-03319-w (PMC11100118; doi:10.1186/s12916-024-03319-w)
Supplement: Supplementary file 2 — Additional file 2: Table S2. Information Sheet on Genes Associated with Osteosarcoma Lung Metastasis Score. [file 12916_2024_3319_MOESM2_ESM.docx]

Table S2 Information Sheet on Genes Associated with Osteosarcoma Lung Metastasis Score

| **Gene** | **PMID** | **Author** |
| --- | --- | --- |
| KDM6B | 33664867 | Yuhang Jiang |
| CBX4 | 32111827 | Xin Wang |
| RAB22A | 33568623 | Li Zhong |
| PTEN | 29807230 | Chao Yu |
| KEAP1 | 29569716 | Huaiyuan Xu |
| SKP2 | 30250282 | Yidan Zhang |
| IMP3 | 36937398 | Shuangwu Dai |
| ZEB1 | 28694763 | Yi Deng |
| EZH2 | 37120706 | Zhiyu Chen |
| EZR | 35012433 | Qin Yao |
| TRAF4 | 25700355 | Weitao Yao |
| COPS3 | 17366602 | Taiqiang Yan |
| PRAME | 22390931 | Pingxian Tan |
| FASN | 30931932 | Tianhao Sun |
| HMGB1 | 36801636 | Changhe Hou |
| CNN3 | 32667904 | Fei Dai |
| MYH9 | 27262074 | Wei Zhou |
| ACTN4 | 30879239 | Qingshan Huang |
| SPAG5 | 32668328 | Zhiyun Li |
