# Supplementary material for: Single-cell and spatial transcriptomics reveal metastasis mechanism and microenvironment remodeling of lymph node in osteosarcoma
Source: BMC Med. 2024 May 17;22:200. doi: 10.1186/s12916-024-03319-w (PMC11100118; doi:10.1186/s12916-024-03319-w)
Supplement: Supplementary file 3 — Additional file 3: Table S3. The siRNA sequences. [file 12916_2024_3319_MOESM3_ESM.docx]

Table S3 The siRNA sequences

| Gene name | sequence |
| --- | --- |
| siETS2-01  siETS2-02  siETS2-03 | Sense：CGCCAACUGUGAAUUGCCUUUTT  Antisense：AAAGGCAAUUCACAGUUGGCGTT  Sense：CCUGACUUUGUGGGUGACAUUTT  Antisense：AAUGUCACCCACAAAGUCAGGTT  Sense：CCAACCAUGUCUUUCAAGGAUTT  Antisense：AUCCUUGAAAGACAUGGUUGGTT |
| si-IBSP-01  si-IBSP-02  si-IBSP-03 | Sense：GCCUGUGCUUUCUCAAUGAAATT  Antisense：UUUCAUUGAGAAAGCACAGGCTT  Sense：GAGACUUCAAAUGAAGGAGAATT  Antisense：UUCUCCUUCAUUUGAAGUCUCTT  Sense：GAGAAGGAAAGUGAUGAAGAATT  Antisense：UUCUUCAUCACUUUCCUUCUCTT |
| Negative Control | Sense：UUCUCCGAACGUGUCACGUTT  Antisense：ACGUGACACGUUCGGAGAATT |
