# Supplementary material for: Single-cell and spatial transcriptomics reveal metastasis mechanism and microenvironment remodeling of lymph node in osteosarcoma
Source: BMC Med. 2024 May 17;22:200. doi: 10.1186/s12916-024-03319-w (PMC11100118; doi:10.1186/s12916-024-03319-w)
Supplement: Supplementary file 4 — Additional file 4: Table S4. Sequence of the primer of IBSP and ETS2. [file 12916_2024_3319_MOESM4_ESM.docx]

| Gene | sequence |
| --- | --- |
| IBSP-F | CACTGGAGCCAATGCAGAAGA |
| IBSP-R | TGGTGGGGTTGTAGGTTCAAA |
| ETS2-F | CAGTCTGGTGAACGTGAATCTG |
| ETS2-R | CGGAGGTGAGGTGTGAATTTT |

Table S4 Sequence of the primer of IBSP and ETS2.
