# Supplementary figures and images for: Single-cell and spatial transcriptomics reveal metastasis mechanism and microenvironment remodeling of lymph node in osteosarcoma
Source: BMC Med. 2024 May 17;22:200. doi: 10.1186/s12916-024-03319-w (PMC11100118; doi:10.1186/s12916-024-03319-w)

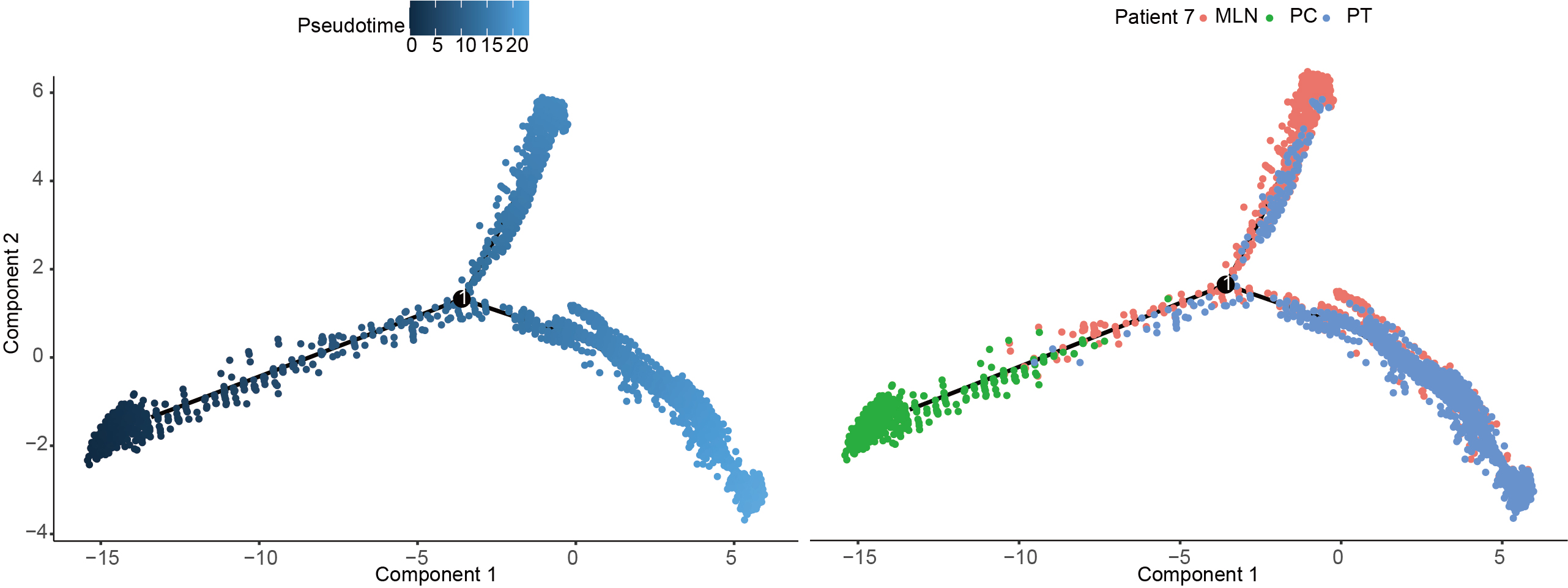

Supplement: Supplementary file 5 — Additional file 5: Figure S1. The pseudotime analysis for Patient 7 [file 12916_2024_3319_MOESM5_ESM.jpg]

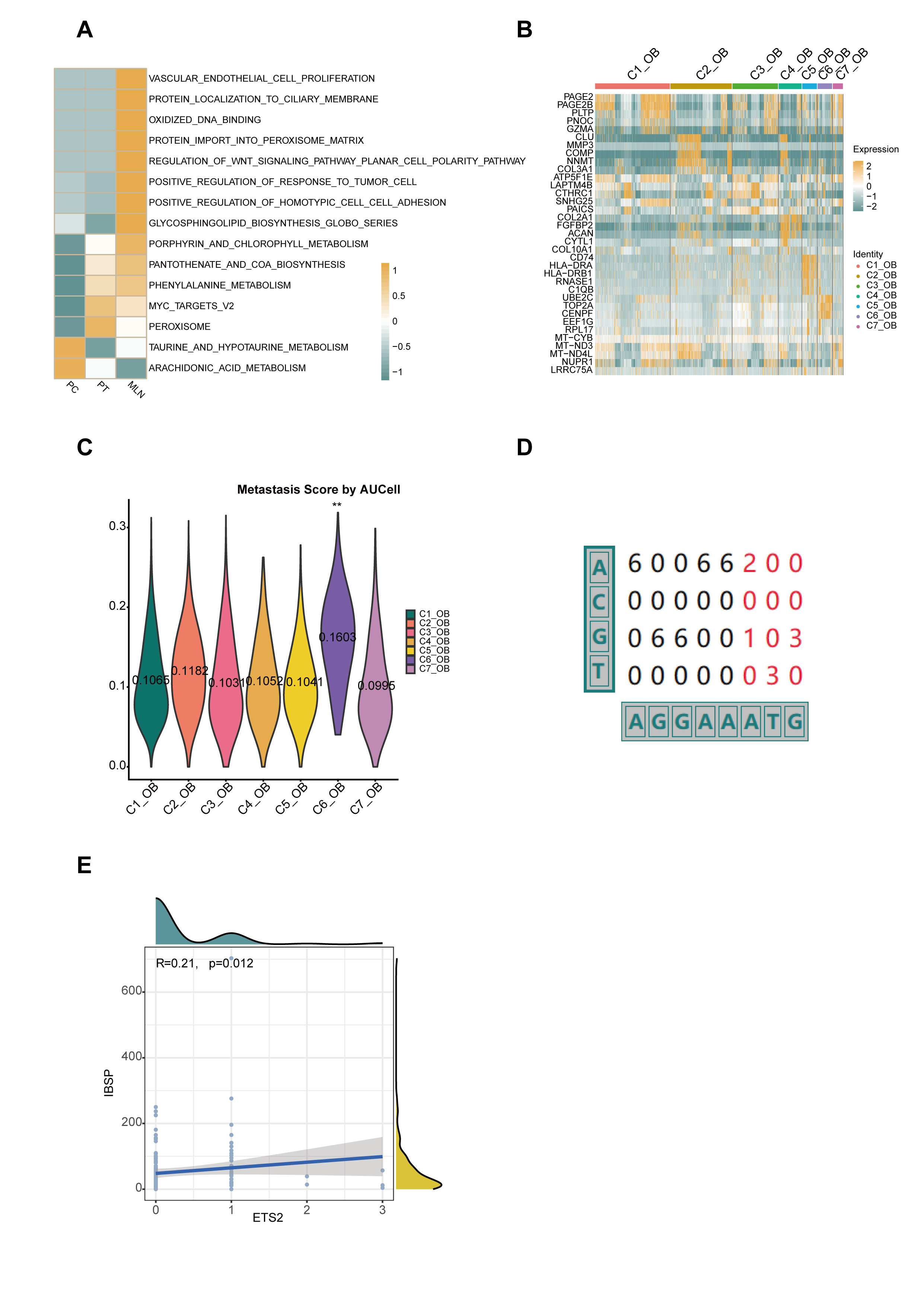

Supplement: Supplementary file 6 — Additional file 6: Figure S2. (A) The heatmap of GSVA analysis of OB from different tissue sources (B) The heat map of differential genes in clusters of OB cells. (C) Metastasis scores significantly higher in C6 cluster cells by AUCell. (D) Sequences predicted by the promo database for possible binding of IBSP with ETS2. (E) Correlation analysis of IBSP and ETS2 expression in OB cells. (***p < 0.001) [file 12916_2024_3319_MOESM6_ESM.jpg]

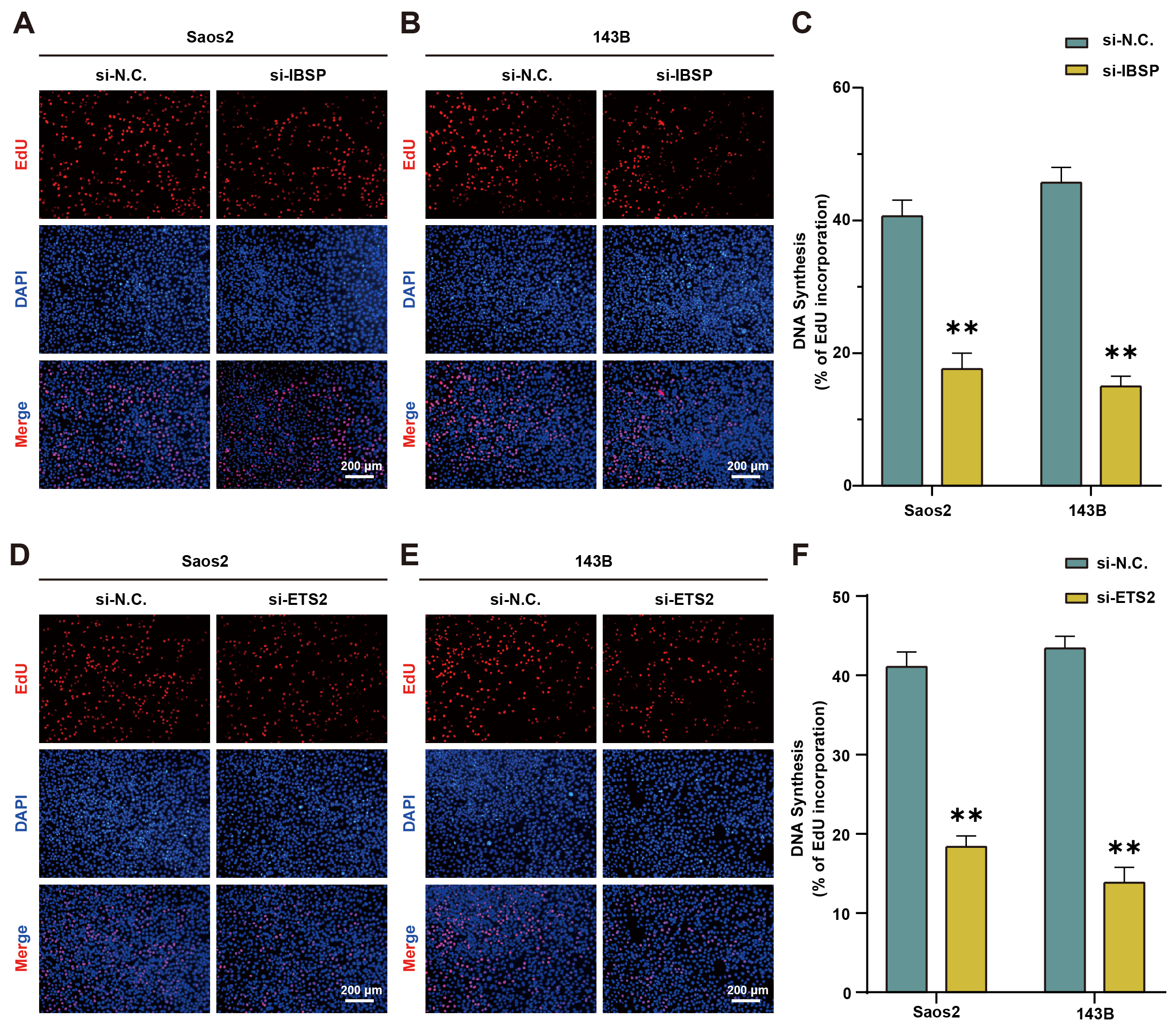

Supplement: Supplementary file 7 — Additional file 7: Figure S3. (A-C) Significantly reduced admixture of EdU in si-IBSP cells compared to controls in two osteosarcoma cell lines. (D-F) Significantly reduced admixture of EdU in si-ETS2 cells compared to controls in two osteosarcoma cell lines. [file 12916_2024_3319_MOESM7_ESM.jpg]

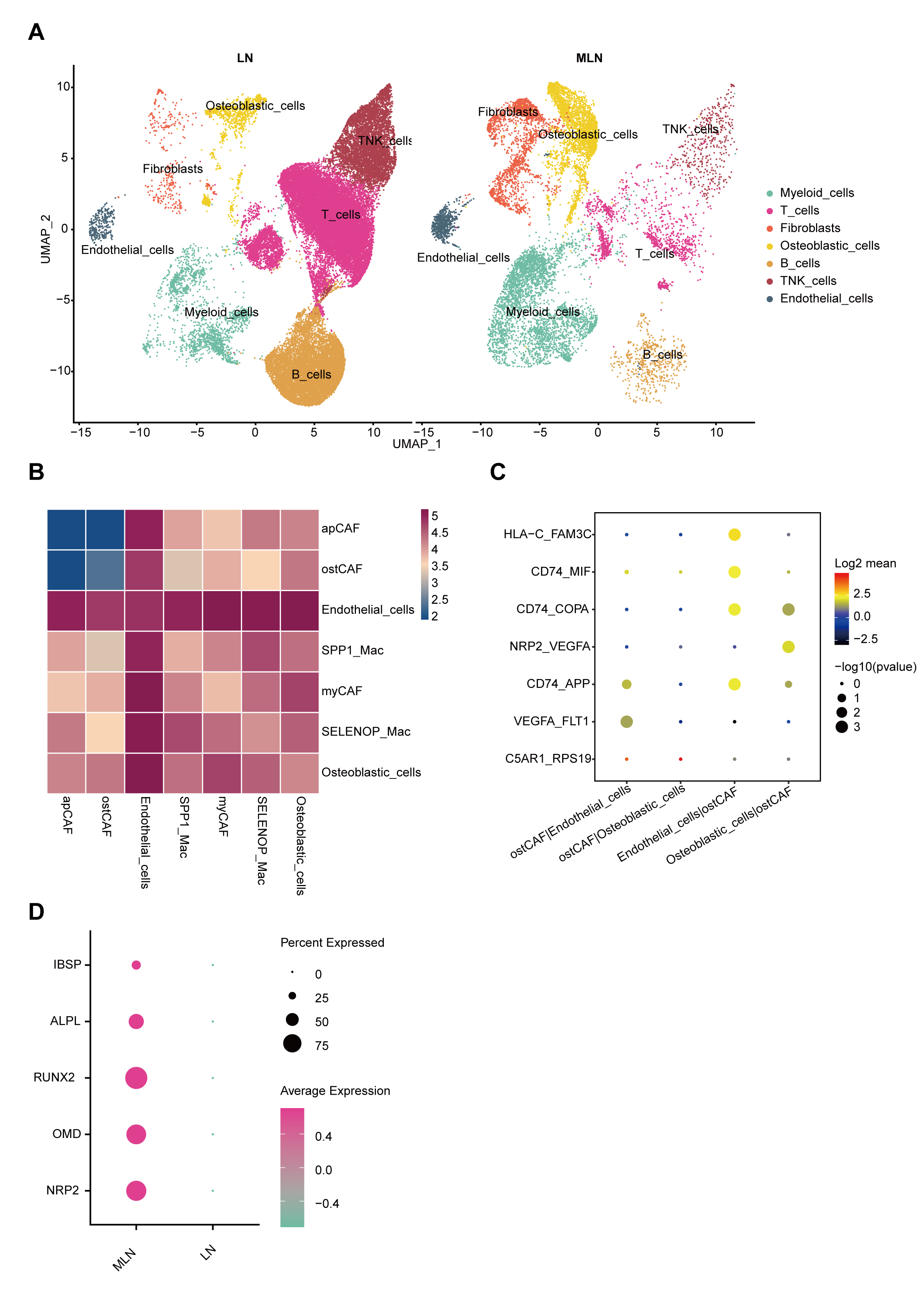

Supplement: Supplementary file 8 — Additional file 8: Figure S4. (A) The UMAP plot of cell clusters categorized by LN and MLN. (B) The heat map of the strength of cellular interaction relationships between myeloid cells and OBs. (C) The bubble plot of the ligand-receptor relationship between myeloid cells and OBs. (D) The bubble plot of OB markers expression in LN and MLN. [file 12916_2024_3319_MOESM8_ESM.jpg]

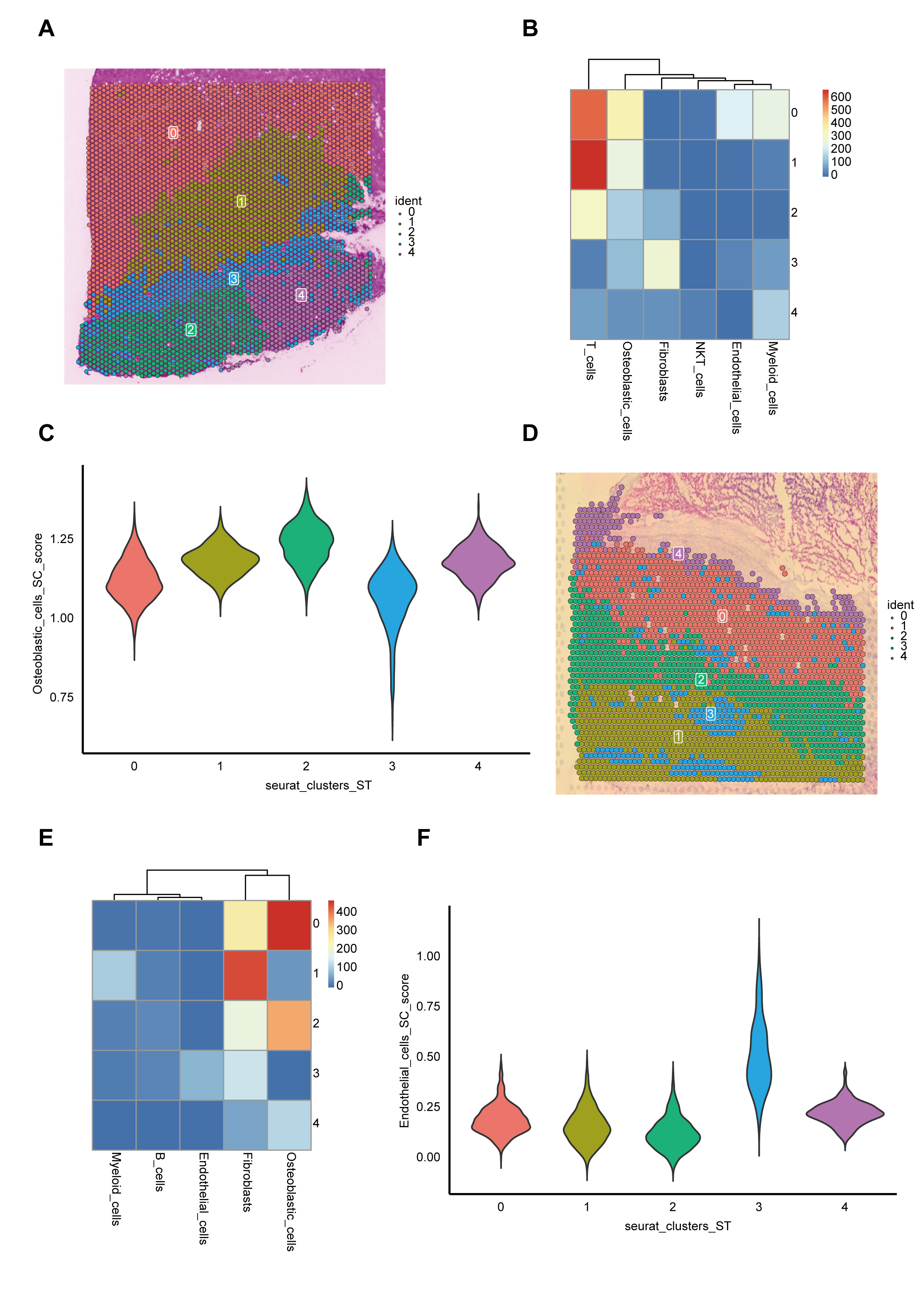

Supplement: Supplementary file 9 — Additional file 9: Figure S5. (A) The spatial location of initial subgroups of patient 9. (B) Inverse convolution was used to define subgroups 0 and 1 as T cells, subgroup 3 as fibroblasts, and subgroup 4 as myeloid cells in patient 9. (C) Subgroup 3 was identified as osteoblastic cells by AddModuleScore. (D) The spatial location of initial subgroups of patient 9. (E) Inverse convolution was used to define subgroups 0 and 2 as osteoblastic cells and subgroup 1 as fibroblasts in patient 9. (F) Subgroup 3 was identified as endothelial cells by AddModuleScore. [file 12916_2024_3319_MOESM9_ESM.jpg]
